# Green synthesis of collagen nanoparticles by *Streptomyces xinghaiensis* NEAA-1, statistical optimization, characterization, and evaluation of their anticancer potential

**DOI:** 10.1038/s41598-024-53342-3

**Published:** 2024-02-08

**Authors:** Asmaa A. El-Sawah, Noura El-Ahmady El-Naggar, Heba E. Eldegla, Hoda M. Soliman

**Affiliations:** 1 Botany Department, Faculty of Science, Mansoura University, Mansoura, Egypt; 2 Department of Bioprocess Development, Genetic Engineering and Biotechnology Research Institute, City of Scientific Research and Technological Applications (SRTA-City), New Borg El-Arab City, 21934 Alexandria Egypt; 3 Medical Microbiology and Immunology Department, Faculty of Medicine, Mansoura University, Mansoura, Egypt

**Keywords:** Biomaterials, Nanoparticles, Applied microbiology, Biopolymers

## Abstract

Collagen nanoparticles (collagen-NPs) are promising biopolymeric nanoparticles due to their superior biodegradability and biocompatibility. The low immunogenicity and non-toxicity of collagen-NPs makes it preferable for a wide range of applications. A total of eight morphologically distinct actinomycetes strains were newly isolated from various soil samples in Egypt. The cell-free supernatants of these strains were tested for their ability. These strains' cell-free supernatants were tested for their ability to synthesize collagen-NPs. Five isolates had the ability to biosynthesize collagen-NPs. Among these, a potential culture, *Streptomyces* sp. NEAA-1, was chosen and identified as *Streptomyces xinghaiensis* NEAA-1 based on 16S rRNA sequence analysis as well as morphological, cultural and physiological properties. The sequence data has been deposited at the GenBank database under the accession No. OQ652077.1. Face-centered central composite design (FCCD) has been conducted to maximize collagen-NPs biosynthesis. Maximum collagen-NPs was 8.92 mg/mL under the condition of 10 mg/mL of collagen concentration, initial pH 7, incubation time of 48 h and temperature of 35 °C. The yield of collagen-NPs obtained via FCCD optimization (8.92 mg/mL) was 3.32-fold compared to the yield obtained under non-optimized conditions (2.5 mg/mL). TEM analysis of collagen-NPs showed hollow sphere nanoscale particles with mean of 32.63 ± 14.59 nm in diameter. FTIR spectra showed major peaks of amide I, amide II and amide III of collagen and also the cell-free supernatant involved in effective capping of collagen-NPs. The biosynthesized collagen-NPs exhibited anti-hemolytic, antioxidant and cytotoxic activities. The inhibitory concentrations (IC_50_) against MCF-7, HeP-G2 and HCT116 cell lines were 11.62 ± 0.8, 19.60 ± 1.2 and 41.67 ± 2.2 µg/mL; respectively. The *in-vivo* investigation showed that collagen-NPs can suppress Ehrlich ascites carcinoma (EAC) growth in mice. The collagen-NPs/DOX combination treatment showed considerable tumor growth suppression (95.58%). Collagen-NPs evaluated as nanocarrier with a chemotherapeutic agent, methotrexate (MTX). The average size of MTX loaded collagen-NPs was 42.73 ± 3.5 nm. Encapsulation efficiency percentage (EE %) was 48.91% and drug loading percentage (DL %) was 24.45%.

## Introduction

Nanoparticles (NPs) are widely used in a variety of fields. Unique physical characteristics of NPs include their nature, bioavailability, biocompatibility, effective drug delivery, bioactivity, tumor targeting, and anti-inflammatory properties increased the use of NPs in the applied microbiological, and biotechnological applications including biomedical, food, agriculture, etc.^1^. Nanomedicines are being utilized more frequently for drug delivery because of their numerous benefits, such as tissue targeting, controlled release, enhanced drug permeability and solubility, lower toxicity, increased effectiveness, and improved safety. There have been some worries regarding probable genotoxicity and carcinogenicity due to the possible long-term accumulation of non-biodegradable nanoparticles (gold, silver, carbon nanotubes, graphene, silica and titania etc.) in tissue and organs. On the other hand, several naturally occurring biodegradable materials, such as polysaccharides like chitosan and cellulose, proteins, lipids, and peptides have been utilized to fabricate different kinds of nanostructures^2^.

Biological protein-based nanoparticles come in a variety of forms, including collagen, keratin, silk, soy, and elastin. These particles have the benefits of being bioavailable, biodegradable, and relatively inexpensive^3^. The most prevalent protein in the human body, collagen, is present in the bones, muscles, skin, and other connective tissues. The famous protein collagen has been extensively used in medical applications, such as the creation of microspheres and microneedles for the administration of drugs^4^, making protein delivery tablets and pellets^5^, cancer treatment^6^, making gels and liposomes to deliver medicines for a longer time^7^, and collagen shields in ophthalmology^8^. Due to their tiny size, broad surface area, and absorptive capacity, collagen-NPs can disperse in water to generate colloidal solutions. Collagen-NPs also improve cell retention, are easily sterilized, are thermally stable, and lessen the effects of hazardous byproducts produced during breakdown^9^. Due to their superior biodegradability and biocompatibility, high contact surface, low antigenicity, decreased toxicity, and high cationic-charge density potential resulting from their high number of amino groups, collagen-NPs are more useful than other naturally occurring and synthetic polymeric NPs^10^.

Various chemical, physical, and self-assembly procedures, including nano spray drying, emulsification, phase separation, complex coacervation, desolvation, and many other methods were used to produce nano collagen. Also, nano emulsion, electrospinning, milling and electrospray deposition techniques were used to produce nano collagen fibers^11^. While chemical and physical synthesis techniques are more widely used to create NPs, their biomedical applications, particularly in the clinical domain, are severely restricted by the use of hazardous substances. Therefore, in order to increase the number of biomedical uses for NPs, it is critical to create trustworthy, safe, and environmentally acceptable procedures for their synthesis. Biological approaches for producing nanoparticles are quick, affordable, and environmentally benign. Because of this, a wide variety of plants, microbes, including algae, bacteria, fungi, and actinomycetes, are used to synthesize nanoparticles by using their enzymes, proteins, DNA, lipids, carbohydrates, etc. in addition to using less energy, with avoiding toxic substances and hazardous byproducts^12^.

Actinomycetes have been a resource for the synthesis of nanoparticles and bioactive chemicals in recent decades. In modern nanotechnology, actinomycetes are also employed to synthesize different nanoparticles that are utilized as biological control agents or nanofertlizers in sustainable agriculture practices^13^.

Actinomycetes possess the ability to produce both the extracellular and intracellular nanoparticles^13^. Gold nanoparticles were produced using the cell-free supernatant of *Streptomyces flavolimosus* as a bioactive reducing and stabilizing agent^14^. In an innovative microbial-based approach, chitosan nanoparticles were synthesised using *Streptomyces microflavus*^15^. Biofabrication of silver nanoparticles was also accomplished with the help of actinomycetes^16–19^.

The aims of this study were to determine the strain that was more efficient in biosynthesizing collagen-NPs out of eight newly isolated strains, to identify the selected strain, to use the cell-free supernatant of *Streptomyces xinghaiensis* NEAA-1 for biosynthesis of collagen-NPs, to determine the ideal collagen concentration, pH, temperature, and incubation time that maximize the biosynthesis of collagen-NPs using a face-centered central composite design, to assess the collagen-NPs' cytotoxic, antioxidant, and anti-hemolytic effects on the MCF-7, HeP-G2, and HCT116 cell lines. The efficacy of collagen-NPs to stop tumor growth in mice bearing Ehrlich ascites carcinoma (EAC) and utilization of collagen-NPs as nanocarrier with methotrexate (MTX) were also examined.

## Materials and methods

**Figure 1:**
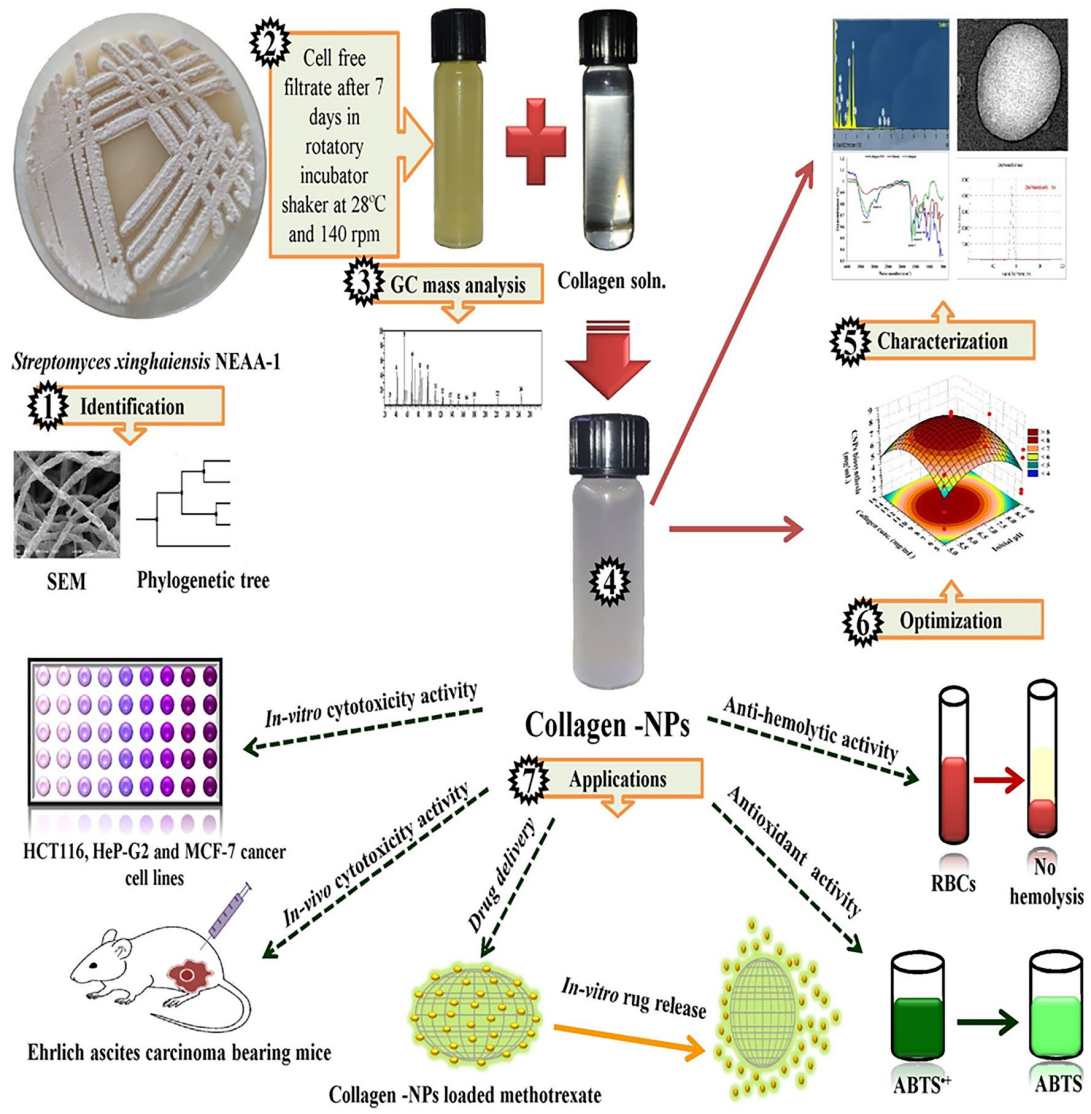
Schematic diagram of the research design framework of this study.

A schematic representation of the research design framework of this study has been provided in Fig. 1.

### Microorganisms and culture conditions

In this research, eight newly isolated strains (coded as NEAA-1, NEAA-J, NEAA-1F, NEAA-26, NEAA-29, NEAA-6B, NEAA-82, NEAA-U3) kindly provided by the second author Prof. Noura El-Ahmady El-Naggar. The eight strains were preserved on slopes containing starch-nitrate agar medium^20^ composed of (g/L) agar, 20; soluble starch, 20; MgSO_4_.7H_2_O, 0.5; KNO_3_, 2; NaCl, 0.5; K_2_HPO_4_, 1; FeSO_4_.7H_2_O, 0.01; and 3; CaCO_3_ in one liter of distilled water, which was then incubated for seven days at 30 °C. For further research, the *Streptomyces* isolates were kept as spore suspensions in 20% (v/v) glycerol solution at − 20 °C.

### Preparation of the cell-free supernatant

The eight strains have been screened for their ability to biosynthesize collagen-NPs. Each strain was cultured on starch nitrate agar media in petri dishes. Three disks measuring 9 mm in diameter from the previously prepared culture were taken and inoculated in Erlenmeyer flasks (250 mL) containing 50 mL of the following medium. In 1 L of distilled water, combine soluble starch, 20; MgSO_4_, 0.5; KNO_3_, 1; NaCl, 0.5; K_2_HPO_4_, 0.5; FeSO_4_·7H_2_O, 0.1; and 0.3 yeast extract. The flasks were incubated for 5–7 days in a rotatory incubator shaker at 30 °C and 150 rpm. Following centrifugation at 8,000 rpm for 15 min at 4 °C to separate the cell-free supernatant from the mycelium. The cell-free supernatants have been lyophilized for characterization by GC–MS analysis using a gas chromatograph (Shelton, CT, USA).

### Extracellular biosynthesis of collagen-NPs

For the biosynthesis of collagen-NPs, 1 mL of 10 mg/mL of pure marine collagen type I solution (MM Ingredients Ltd, UK) was added to 9 mL of freshly mycelium free supernatant (pH was adjusted to 7) and incubated at 35 °C for 48 h. The biosynthesis was observed as white turbidity appearance and confirmed by TEM analysis.

### Cultural and morphology characteristics of *Streptomyces *sp*.* strain NEAA-1

After 14 days of incubation at 30 °C on a medium of starch nitrate agar, spore chain morphology and spore surface ornament of the most effective isolate (*Streptomyces* sp*.* strain NEAA-1) have been examined at Electron Microscope Unit of Mansoura University, Egypt using (JEOL JSM 6510 lv). Pigmentation of substrate mycelia, colour of the aerial mass spore and releasing of diffusible pigments also have been observed on ISP 1 (tryptone-yeast extract agar), ISP 2 (yeast-malt extract agar), ISP 3 (oatmeal agar), ISP 4 (inorganic salt starch agar), ISP 5 (glycerol-asparagine agar), ISP 6 (peptone-yeast extract iron agar), ISP 7 (tyrosine agar) as termed by Shirling & Gottlieb^21^; for 14 days, all plates were incubated at 30 °C.

### Physiological characteristics of *Streptomyces *sp*.* strain NEAA-1

Carbon sources utilization has been tested on basal mineral salts agar and production of melanin has been examined on ISP 1, ISP 6 and ISP 7 based on the methods of Shirling & Gottlieb^21^. According to the study of Tresner et al*.*^22^, the growth in the existence of NaCl was detected. Gelatin liquefaction was detected by^20^ method. Milk coagulation was detected by the method of Cowan & Steel^23^. The strain was streaked onto a starch nitrate agar medium plate containing 2% soluble starch and cultured for 7 days at 28 °C in order to identify the strain's ability to break down starch (production of α-amylase). After incubation, the plate was then stained with Gram's iodine solution, according to the study of Mishra & Behera^24^.

### Sequence alignment, phylogenetic analysis, and 16S rRNA sequencing of *Streptomyces *sp*.* strain NEAA-1

16S rRNA of *Streptomyces* sp*.* strain NEAA-1 was isolated based on Sambrook et al*.*^25^. 16S rRNA was amplified by PCR performed based on El-Naggar et al*.*^16^ methods. Resulted sequence has taken the accession number OQ652077.1 in the GenBank database. The 16S rRNA gene sequence (partial) of *Streptomyces* sp*.* strain NEAA-1 has been aligned with other species of *Streptomyce*s genus with the corresponding 16S rRNA sequences retrieved from the GenBank, EMBL, DDBJ and PDB databases by using BLAST program (https://blast.ncbi.nlm.nih.gov/Blast.cgi;^26^). Using the MEGA 11 software program and the 16S rRNA gene sequences of *Streptomyces* sp*.* strain NEAA-1 and similar organisms, a phylogenetic tree was created using the neighbor-joining algorithm approach^27^.

### Characterization of collagen-NPs using spectroscopic investigation

The biosynthesized collagen-NPs were investigated after 48 h and the UV–Vis absorbance was recorded between 200 and 800 nm using ATI Unicam 5625 UV/VIS Vision Software V3.20.

### Transmission electron microscopy (TEM)

The morphology and size of the collagen-NPs was detected using ultra high resolution of TEM (JEOL-JEM-100 CXII instrument).

### Scanning electron microscopy (SEM)

Freeze-dried thin film of the collagen-NPs was characterized by SEM using JEOL JSM 6510 lv.

### Energy-dispersive X-ray (EDX)

The elemental composition identification of the freeze-dried collagen-NPs carried out by EDX using Oxford X-Max 20.

### Fourier-transform infra-red (FTIR)

The functional groups presented in the freeze-dried collagen-NPs were detected using Thermo Scientific Nicolet iS10 FT-IR spectrometer.

### X-ray diffraction analysis (XRD)

An X-ray diffractometer (Bruker D2 PHASER 2nd Gen) was used to record the XRD spectra of collagen powder and collagen-NPs at room temperature. Monochromatic Cu/K radiations (λ = 1.5405) were used to irradiate the samples at a scanning rate of 2 min^−1^, with diffraction angles ranging from 10° to 80°.

### Zeta potential distribution

Evaluation of the net surface charge of the biosynthesized collagen-NPs was achieved by Zeta potential analysis using Zeta sizer nano ZS90, Malvern Instruments Ltd., U.K.

### Thermogravimetric analysis (TGA) and differential scanning calorimetry (DSC)

Using a thermo-analyzer of type 50-H, collagen and collagen-NPs TGA analyses were accomplished. For TGA analyses, temperatures ranging from room temperature to 800 °C were applied to both samples in increments of 10 °C min^−1^. Samples of freeze-drying collagen and collagen-NPs weighing about 2.1 mg and 1.7 mg, respectively, were examined in a nitrogen environment at a flow rate of 30 mL/min. The proportion of weight loss relative to temperature was displayed in the diagram. The pyrolysis pattern for both collagen and collagen-NPs were examined using DSC analysis. The scans were conducted at temperatures ranging from ambient temperature to 500 °C. The diagram depicted heat flow vs temperature.

### Experimental design for face central composite design (FCCD)

The influence of 4 variables, pH level (X_1_), temperature (X_2_), incubation time (X_3_), and collagen content (X_4_) on the production of collagen nanoparticles (collagen-NPs) has been studied using an FCCD model with 30 runs and three replicates. At three different levels (-1, 0, 1), each variable in the design have been studied. For experimental design, the central values (zero level) repeated six times and were: 10 mg/mL collagen soln., incubation time 48 h, pH level 7 and temperature 35 °C. The data of experiments have been fitted with the second polynomial regression equation:

1 $$Y = \beta_{0} + \sum\limits_{i} {\beta_{i} X_{i} + \sum\limits_{ii} {\beta_{ii} X_{i}^{2} } } + \sum\limits_{ij} {\beta_{ij} X_{i} X_{j} }$$

where Y is the predicted collagen-NPs and *β*_i_, *β*_ii_, *β*_ij_ are the linear, quadratic, and interaction terms, respectively. *β*_0_ is a constant. *X*_*i*_ and *X*_j_ are the coded levels were symbolized as X_1_, X_2_, X_3_ and X_4_.

### Statistical analysis

Applying Design Expert® 12 software for Windows (version 12; Stat-Ease Inc., USA), (https://www.statease.com/software/design-expert/), optimization and experimental data analysis were carried out. By using Version 8.0, StatSoft Inc., Tulsa, USA, STATISTICA software, the 3D surface graph of two different variables was plotted against the production of collagen-NPs (https://www.statsoft.de/de/software/statistica).

### Anti-hemolytic activity of collagen-NPs

A blood specimen was taken from the cardiac puncture of rats and put in heparin tubing. The buffy coat was washed three times with 10 volumes of NaCl (0.15 M) after the erythrocytes were separated from the plasma. During the final wash, erythrocytes were centrifuged at 2500 rpm/min for 10 min to obtain packed erythrocyte cells. Peroxyl radicals aided hemolysis in this test method. The same volume of 200 mM 2, 2-azobis (2-amidinopropane) dihydrochloride (AAPH) in PBS containing collage-NPs was mixed with a 10% suspension of phosphate buffered saline pH 7.4 (PBS) containing erythrocytes at various concentrations. The mixture was gently shaken for two hours at 37 °C, diluted with 8 volumes of PBS, and then centrifuged at 1500 rpm for ten minutes. At 540 nm, the supernatant absorbance was measured. The mixture had the same treatment with 8 volumes of distilled water. After centrifuging, the absorbance of the supernatants was measured at 540 nm together with H_2_O for complete hemolysis. L-ascorbic acid was the positive control that was employed. The Eq. (1-A/B) × 100% was used to compute the hemolysis percentage. The results were presented as mean and standard deviation. A positive control was employed, which was vitamin C (L-ascorbic acid).

### In vitro cytotoxicity of collagen-NPs on cancer and normal cell lines

The 3-(4, 5-Dimethyl thiazol-2yl)-2, 5-diphenyl tetrazolium bromide (MTT) assay (colorimetric approach) was used to test the collagen-NPs' in vitro cytotoxicity against normal and cancer cell lines. Cell lines for WI38 (human lung fibroblast), WISH (human amnion), HCT116 (human colorectal carcinoma), HeP-G2 (human liver cancer), and MCF-7 (mammary gland breast cancer) were received from ATCC via Holding company for Biological Products and Vaccines (VACSERA), Cairo, Egypt. Due to the existence of NAD-dependent mitochondrial dehydrogenase in viable cells, the appearance of formazan's purple color is directly proportional to the quantity of viable cells^28^. The cells have been cultivated in RPMI-1640 media containing 10% fetal bovine serum, 100 units/mL penicillin, and 100 µg/mL streptomycin antibiotics at 37 °C in a 5% CO_2_ incubator. Trypsin-ethylene diamine tetra acetic acid (EDTA) has been used to separate monolayer cells for single cell suspensions. The viable cells have been estimated using a hemocytometer. In 96-well plates, 100 µL of cell suspensions have been seeded in each well, retaining the density of 10,000 cells/well, and then incubation has been applied for 48 h at 37 °C with 5% CO_2_, 100% relative humidity and 95% air for attachment of cells to the well’s bottoms. Doxorubicin (DOX) used as standard and collagen-NPs were utilized to treat the cells at various concentrations (1.56, 3.125, 6.52, 12.5, 25, 50, and 100 µg/mL), and collagen-NP concentrations were first put through a 0.45-m filter syringe. After 24 h of incubation at 37 °C with 5% CO_2_, 100% relative humidity, and 95% air were used. To guarantee the correctness of the results, the study has been duplicated. Each well received 5 mg/mL phosphate buffered saline and 20 µL of the yellow MTT solution. For MTT reduction, the plates were incubated for 4 h at 37 °C. Finally, 100 µL of DMSO were added to the resulting purple formazan crystals, and an EXL 800 plate reader was used to measure the absorbance at 570 nm.

The percentage of cytotoxicity has been determined by:

2 $${\text{Viability}}\,{\text{\% }} = \left( {{\text{Test}}\,{\text{OD}}/{\text{Control}}\,{\text{OD}}} \right) \times 100$$

3 $${\text{Cytotoxicity}}\,{\text{\% }} = 100 - {\text{Viability\% }}$$

### ABTS^+^ radical scavenging (anti-oxidant) activity assay

In order to measure the antioxidant activity of collagen-NPs, 3 mL of MnO_2_ (25 mg/mL) solution was combined with 2 mL of (60 M) ABTS (2, 2′-Azino-bis (3-ethylbenzthiazoline-6-sulfonic acid) solution. In 5 mL of PBS (pH 7, 0.1 M), both had previously been prepared. After the combination was shaken, centrifuged, and filtered to produce a green–blue solution (ABTS^+^ solution), the absorbance (A control) was adjusted to a value of approximately 0.5 at 734 nm. Then, combine 1 mL of the ABTS^+^ solution with 1 mL of collagen-NPs (500 µg/mL) and let the mixture sit for 30 min at room temperature before measuring the absorbance at 734 nm (A _test_) with a microplate reader. The same procedure was used to compare collagen powder. Inhibition was used to express the absorbance (A_test_), which related to the decrease in color intensity. The percentage inhibition is determined as follows: Ascorbic acid (vitamin C) served as the positive control, with the ratio being (A_control_ − A_test_)/A_control_ × 100.

### In vivo Ehrlich apoptosis stimulation by collagen-NPs

The impact of collagen-NPs on the Ehrlich solid tumor growth and apoptosis of has been investigated in order to determine whether collagen-NPs stimulate apoptosis in vivo.

### Statement of ethics

All experiments were carried out in accordance with the applicable laws and regulations. Research Ethics Committee, Faculty of Pharmacy, Mansoura University, Mansoura, Egypt, carried out all experimental protocols.

### Animals

Adult Swiss female albino mice weighing 25–30 g were bought from the Institute of Theodore Bilharz Research in Giza, Egypt. At the Faculty of Pharmacy, Mansoura University, Mansoura, Egypt, they were kept and housed in standard-sized polycarbonate cages with free access to food and water at all times. At the laboratory, the animals were housed at 26 ± 1 °C, a 12-h light/dark cycle, 25 °C, and a relative humidity of 20%.

### Ehrlich solid tumor model

To create solid tumors, all mice were first subcutaneously injected with 5 × 10^5^ EAC cells from Cairo University's National Cancer Institute (NCI), Cairo, Egypt. The solid tumor volumes were roughly 50–100 mm^3^ (day 0) after five days of inoculation, prior to the start of the treatment. The mice were then put into six groups at random. There were six animals per group. Group I served as the control (only EAC-bearing mice); Group II, EAC-bearing mice injected in tumor with collagen-NPs (2 mg/kg/1 day); Group III, EAC-bearing mice injected with the anti-cancer doxorubicin (DOX) alone (2 mg/kg/1 day); Group IV, EAC-bearing mice injected with collagen-NPs and DOX; Group V, EAC-bearing mice injected with collagen(2 mg/kg/1 day); Group VI, EAC-bearing mice injected with collagen and DOX. Following the injection of collagen-NPs and DOX, tumor volumes were measured at the fifth day following tumor induction and then every fifth day for a total of 20 days. Mice were sacrificed by cervical dislocated under anesthesia according to the weight of mouse with its tumor using approximately 1.4 mg thiopental sodium (40 mg/kg) at the conclusion of the trial and treatment (day 21), and tumor lumps were removed, weighed, and stored in buffered formalin solution for histological examination. Vernier calipers can be used to determine the tumor's volume^29^ using the following formula:

4 $${\text{V}} = \left( {{\text{L}} \times {\text{S}}^{2} } \right) \times 0.5$$

where V is the volume of the tumor, L is the diameter of the longest tumor and S is the diameter of the shortest vertical tumor. According to the study of Schirner et al*.*^30^, the effectiveness of the anti-tumor was calculated as:

5 $${\text{Growth}}\,{\text{inhibition}}\,\left( \%  \right) = 100-\left( {\frac{\Delta T}{{\Delta C}} \times 100} \right)$$

where ΔT is the tumor volume change average in the treatment group and ΔC is and the tumor volume change average in the control EAC bearing mice.

Hematoxylin and eosin were used to stain the micrometer-sized slices, which were then viewed under a light microscope.

### Drug loading and encapsulation efficiency

100 mg of collagen-NPs was added to 10 mL of 5 mg/mL methotrexate (MTX) (Hikma Specialized Pharmaceuticals, Badr City, Cairo, Egypt) in order to assess the drug loading (DL%) and encapsulation efficiency (EE%). At 25 °C, the mixture was stirred for 6 h. Through centrifugation at 12,000 rpm for 15 min, MTX-collagen-NPs (MTX-collagen-NPs) were produced as a precipitate. Immediately following centrifugation, the supernatant was collected for use in a UV–Vis to measure absorbance at 377 nm. The process was repeated trice. For quantification of the free MTX concentration in solution, a previously calibration curve was constructed depend on the absorbance changes with different MTX concentration. The percentage of (DL %) and (EE %) were calculated as follows:

6 $${\text{DL}}\,\left( {\text{\% }} \right) = \frac{{{\text{W}}\left( {{\text{total}}} \right){ } - {\text{ W}}\left( {{\text{free}}} \right)}}{{{\text{W}}\left( {{\text{collagen}} - {\text{NPs}}} \right) }} \times 100\%$$

7 $${\text{EE}}\,\left( {\text{\% }} \right) = \frac{{{\text{W }}\left( {{\text{total}}} \right) - {\text{W}}\left( {{\text{free}}} \right)}}{{{\text{W }}\left( {{\text{total}}} \right){ }}} \times 100\%$$

where W _(total)_ refers to the total weight of MTX, W _(free)_ refers to the total weight of free MTX (not loaded) in supernatant, W _(collagen-NPs)_ refers to the total weight of collagen-NPs. The precipitate was washed three times, and then lyophilized for evaluation in vitro drug release.

### In vitro drug release

Phosphate buffer saline (PBS) was used to assess the in vitro release behavior of MTX-collagen-NPs at pH 7.4 (blood pH) and pH 5.5 (acidic intracellular environment). Loading dialysis tubing (MWCO; 12–14 kDa) with 5 mg of MTX-collagen-NPs powder dissolved in 5 mL of PBS (pH 7.4 or pH 5.5). The dialysis tube was submerged in 40 mL of PBS with gentle stirring at 100 rpm at 37 °C and a pH of either 7.4 or 5.5. An aliquot of 3 mL was taken out of the release medium and replaced with an equal aliquot volume of fresh PBS at predefined intervals (0, 3, 6, 8, 12, 24, 48 h). The UV–Vis spectrometer was used to examine the aliquot sample. The process was repeated trice. An equation was used to compute the cumulative release percentage (CR%) of MTX-collagen-NPs at each time interval as follows:

8 $${\text{The}}\,{\text{cumulative}}\,{\text{release}}\,{\text{percentage}}\,\left( {{\text{CR}}\,\% } \right) = \frac{{{\text{Wt}}}}{{{\text{Wi}}}} \times {1}00$$

where W_t_ is released drug weight at time _t_ and W_i_ is MTX-collagen-NPs initial weight.

### Ethics approval and consent to participate

All experiments were carried out in accordance with the applicable laws and regulations. All experimental protocols were approved by a Research Ethics Committee, Faculty of Medicine, Mansoura University, Mansoura, Egypt (Code number: MDP.21.01.54).

The authors confirm that all experiments involving animals were performed in accordance with the ARRIVE (Animal Research: Reporting of In Vivo Experiments) guidelines.

## Result and discussion

In this study, collagen-NPs were biosynthesized from pure marine collagen by the cell-free supernatant of a newly isolated eight strains. The most effective strains for collagen-NPs biosynthesis were *Streptomyces* sp*.* strain NEAA-1 and NEAA-6B yield 2.5 and 2.43 mg/mL of collagen-NPs; respectively. So, *Streptomyces* sp*.* strain NEAA-1 was selected for collagen-NPs biosynthesis and identified.

### Cultural and morphology characteristics of *Streptomyces *sp*.* strain NEAA-1

**Figure 2:**
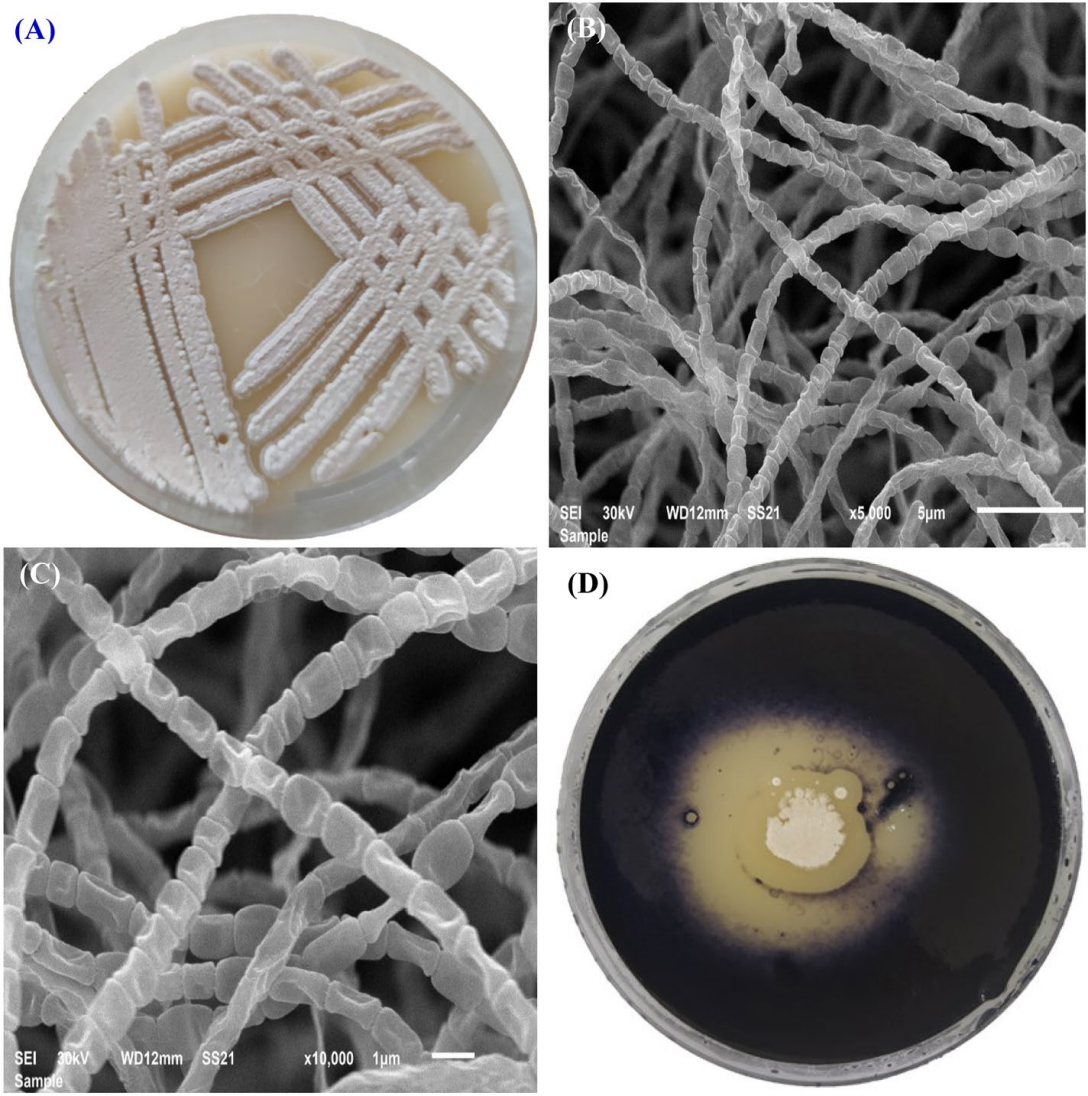
*Streptomyces xinghaiensis* NEAA-1 culture on starch nitrate agar (**A**), and scan electron microscopy photos of *Streptomyces xinghaiensis* NEAA-1 (**B**, **C**), starch hydrolysis.

*Streptomyces* sp*.* strain NEAA-1 is a mesophilic, aerobic, and gram-positive organism. The 7-day-old culture of *Streptomyces* sp*.* strain NEAA-1 grown on starch–nitrate agar^20^ was morphologically observed (Fig. 2A). It was found that both aerial and substrate mycelium were abundant, well-developed, and not fragmented. Aerial mycelium bear rectiflexibiles spore chains; spores are elongated with smooth compact surfaces e and are non–motile (Fig. 2B,C). *Streptomyces* sp*.* strain NEAA-1 mycelia developed well on all of the studied media (tryptone-yeast extract agar, yeast extract-malt extract agar, oatmeal agar, inorganic salt-starch agar, and tyrosine agar), but poorly developed on peptone-yeast extract iron agar and did not grow on glycerol-asparagine agar. On any of the media that were tested (Table 1), no diffusible pigments were found. The mature sporulating aerial mycelium's color ranged from white to gray on the majority of media and was beige on oatmeal agar. On all produced media, the colony's reverse side is a yellowish-brown color; the substrate pigment is not a pH indicator.

**Table 1:** Phenotypic characteristics that separate *Streptomyces* sp*.* strain NEAA-1 from other related *Streptomyces* species.

Characteristic	*Streptomyces* sp. strain NEAA-1	*S. noursei*	S. *xiangtanensis*	*S. xinghaiensis*	*S. oryziradicis*
ISP2					
G	Good	Good	Good	Good	Good
AM	Grayish white	Gray	Chamois	White	−
ISP3					
G	Good	Good	Good	Good	Good
AM	Beige	Light gray	White to gray	White	Grayish brown to pale
ISP4					
G	Good	Good	Good	Good	Good
AM	Gray	Gray	White	White	–
ISP5					
G	None	Good	Good	Good	None
AM	None	Gray	White	Vivid bluish green	None
ISP6					
G	Poor	Good	Good	−	Poor
AM	White	None	−	−	−
ISP7					
G	Good	Good	Good	−	Good
AM	Grayish white	Gray	−	−	−
Spore shape	Elongated with compact surfaces		Cylindrical with smooth-surfaces		
Spore surface	Smooth	Spiny	Smooth		Smooth
Spore chain morphology	Rectiflexibiles		Straight to slightly flexuous		Spiral
Melanin production	−	−	−	−	
Maximum NaCl concentration (%, w/v) tolerance	5	5	3	5	2
Starch hydrolysis	+		+	−	
Gelatin liquefaction	+	+	−	+	
Coagulation of milk	+			+	
Fructose	++	+		+	+
Maltose	++		−	−	−
Lactose	+		−	+	
Glucose	+	+		+	
Sucrose	++	−		−	−
Ribose	+++		−	+	
Xylose	+++			−	+
Galactose	++		−	+	+

+: Positive; − negative; blank cells: no data available; *G* growth; *AM* Arial mycelium.

### Physiological characteristics

Table 1 displays the physiological characteristics of *Streptomyces* sp*.* strain NEAA-1. In peptone-yeast-iron agar, glycerol tyrosine agar, or tryptone-yeast agar, no melanin pigments were produced. Starch hydrolysis (Fig. 2D), coagulation of milk and gelatin liquefaction were positive. The ideal growing temperature was 30 °C, and the pH value was 7.0. The isolate showed tolerance for NaCl up to 5% (w/v). As the only carbon source supply, xylose and ribose, fructose, glucose, sucrose, maltose, glucose and lactose were utilized.

### 16S rRNA gene sequence comparisons and phylogenetic analysis

**Figure 3:**
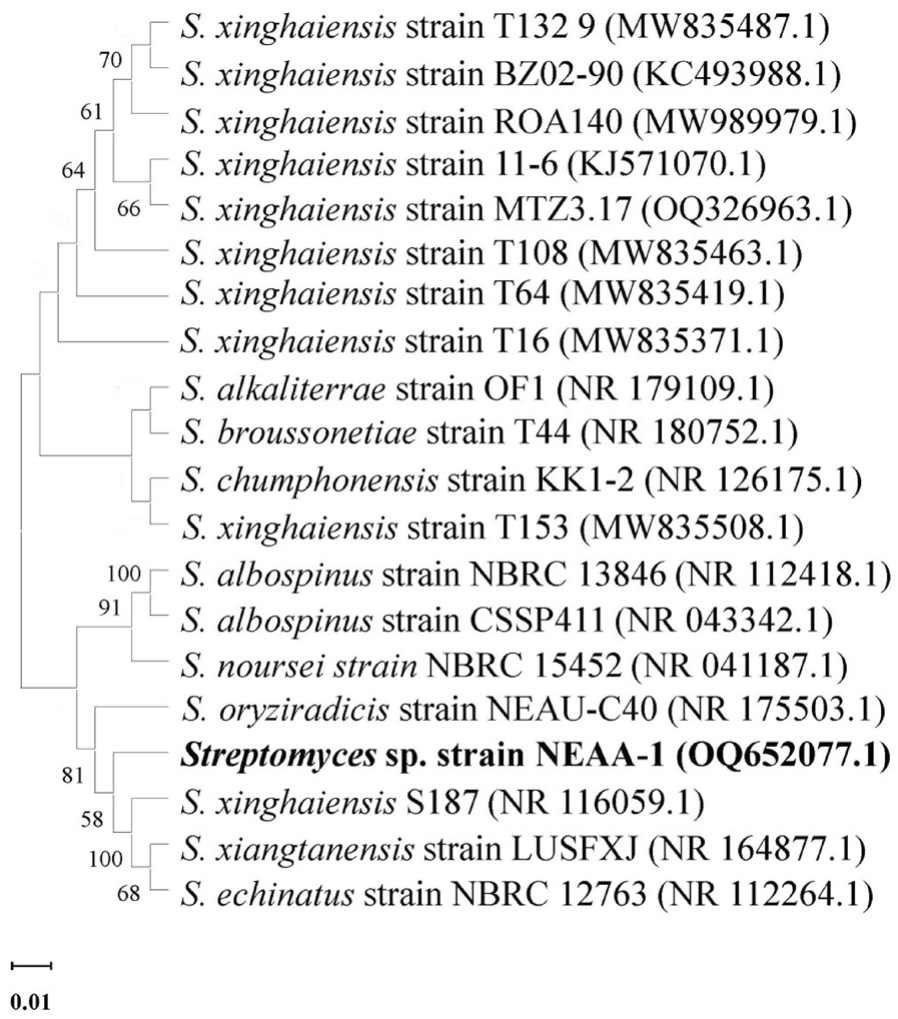
Phylogenetic tree of neighbour-joining constructed on the sequences of 16 S rRNA. Bootstrap values > 50% based on 500 replications are shown at branch nodes. Bar, 0.001 substitutions per nucleotide position, displaying the relationships between other *Streptomyces* species of related with *Streptomyces* sp*.* strain NEAA-1.

In the GenBank/EMBL/DDBJ databases, the partial 16S rRNA gene sequence for *Streptomyces* sp*.* strain NEAA-1 (650 bp) has been deposited under accession No. OQ652077.1 [https://www.ncbi.nlm.nih.gov/nucleotide/OQ652077.1?report=genbank&log$=nucltop&blast_rank=1&RID=G68R6PDV013]”. Using BLAST search (https://blast.ncbi.nlm.nih.gov/Blast.cgi), the NEAA-1 strain was matched with the corresponding 16 rRNA gene sequences of the nearby members of the genus *Streptomyces*^22^. Many species of the genus *Streptomyces* have been found to be comparable to our strain, according to the GenBank database of the BLAST. The neighbor-joining algorithm approach^27^ was used to create the phylogenetic tree (Fig. 3), which demonstrates that *Streptomyces* sp*.* strain NEAA-1 has close phylogenetic link with specific other *Streptomyces* species. According to phylogenetic analyses based on 16S rRNA gene sequences, the isolate based on GenBank/EMBL/DDBJ belonged to subclades with *Streptomyces xinghaiensis* strain S187 (accession No. NR_116059.1) with a similarity of 95.27%, *Streptomyces noursei* strain NBRC15452 (accession No. NR_041187.1) with a similarity of 94.35%, *Streptomyces oryziradicis* strain NEAU-C40 (accession No. NR_175503.1) with a similarity of 94.05% and *Streptomyces xiangtanensis* strain LUSFXJ (accession No. NR_164877.1) with a similarity of 94.05%.

It was determined that *Streptomyces* sp*.* strain NEAA-1 was most closely related to the type strain of *Streptomyces xinghaiensis* strain S187 (accession No. NR_116059.1) with the highest degree of similarity 95.27%^31^ based on the comparative study based on the previous collected data of the morphological, cultural, and physiological characteristics of the isolate. The isolate was thus named as *Streptomyces xinghaiensis* strain NEAA-1. The actinobacteria data for the reference species have been taken from Mo et al*.*^32^; Brown et al*.*^33^; Li et al*.*^34^.

### GC–MS spectral analysis of *Streptomyces xinghaiensis* NEAA-1 cell-free supernatant

**Figure 4:**
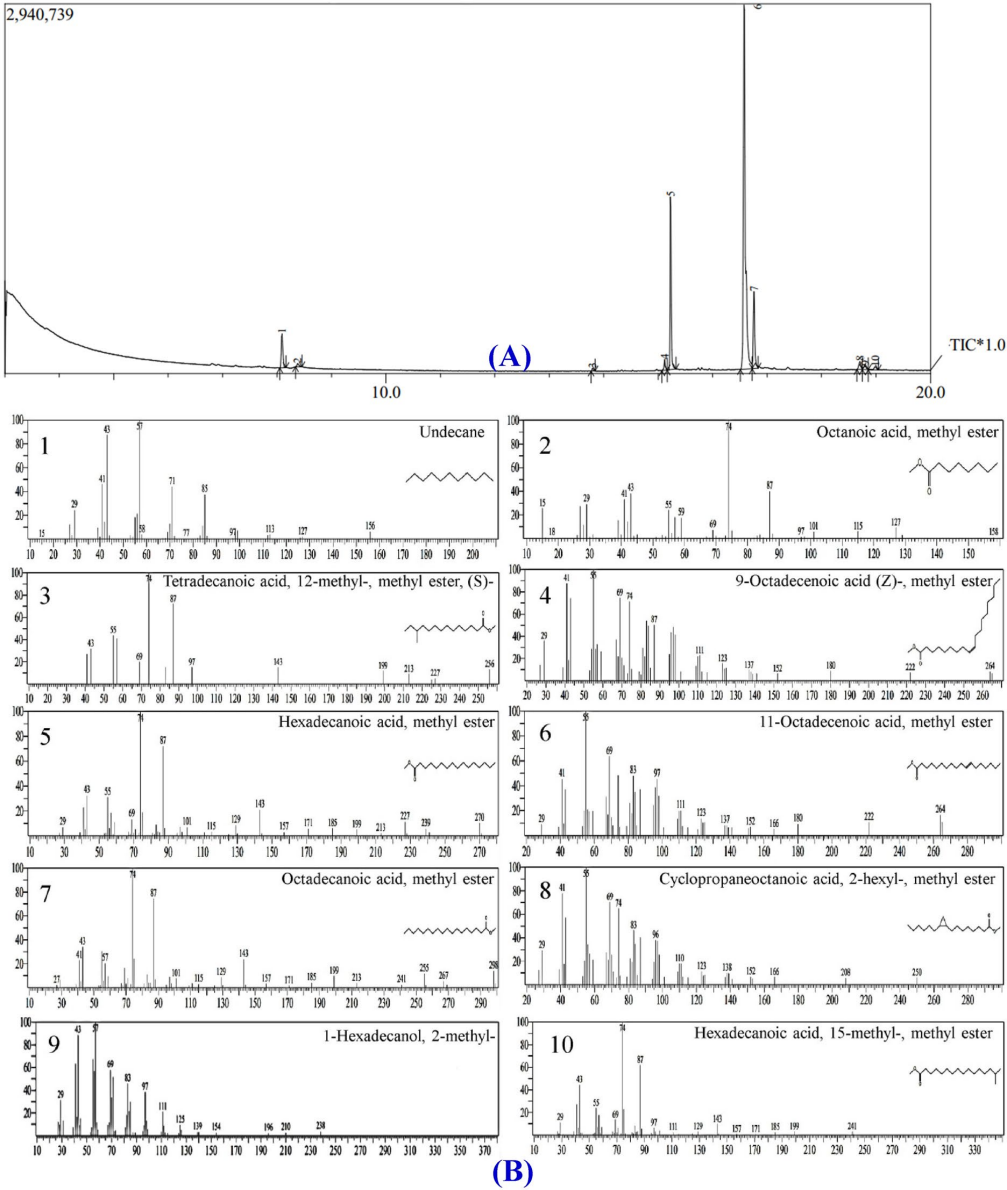
GC–MS chromatogram (**A**) and chemical composition of bioactive components (**B**) identified in the methyl acetate of the cell-free supernatant of *Streptomyces xinghaiensis* NEAA-1.

The GC–MS spectral analysis (Table 2; Fig. 4A,B) of a fraction eluted with methanol: resulted in the identification of ten fatty acids, including nine linear chain saturated fatty acids methyl ester of; 11-octadecenoic acid, octanoic acid (caprylic acid), butanoic acid (butyric acid), pentanoic acid (valeric acid), dodecanoic acid (lauric acid) m, octadecanoic acid (stearic acid), tetradecanoic acid (myristic acid), hexadecanoic acid (palmitic acid) and cyclopropaneoctanoic acid, 2-hexyl. Unsaturated fatty acid; 9-octadecanoic acid (Z) (oleic acid & its isomer elaidic acid) is also present. Three types of alcohols; 2-methyl-1-hexadecanol, 2-isopropyl-5-methyl-1-hexanol and heptacosanol are exhibited. 5-octadecenal is the only aldehyde presented. The most abundant fatty acid was 11-octadecenoic acid, methyl ester which has antimicrobial activity reported by Shoge and Amusan^35^. Octadecanoic acid (otearic acid) methyl ester presented at the third degree then 9-octadecanoic acid (Z) (oleic acid) methyl ester. Palmitic acid, stearic acid and oleic acid showed antioxidant, in vitro inflammatory and antibacterial activity according to the studies of Fratianni et al*.*^36^. Undecane showed a wide spectrum of antimicrobial activities according to the study of Wang et al*.*^37^. Caprylic acid has widespread uses in the health promoting, in cosmetics, other industrial products, dietary supplement, antioxidant for skin in skin products and antimicrobial pesticide for surface sanitization in the food and dairy industry^38^. Butyric acid, myristic acid and lauric acid, methyl ester exhibited antioxidant and antibacterial activities according to the study of Gıdık^39^.

**Table 2:** Bioactive constituents identified in the cell-free supernatant of *Streptomyces xinghaiensis* NEAA-1 using GC–MS analysis.

Mol. wt	Formula	Peak area (%)	Compound	Ret. time (min)
156	C_11_H_24_	9.5	Undecane	8.090
158	C_9_H_18_O_2_	1.2	Octanoic acid, methyl ester (Caprylic acid methyl ester)	8.378
256	C_16_H_32_O_2_	0.73	Tetradecanoic acid, 12-methyl-, methyl ester, (S)-	13.796
296	C_19_H_36_O_2_	1.1	9-Octadecenoic acid (Z)-, methyl ester (Oleic acid, methyl ester)	15.118
270	C_17_H_34_O_2_	28.8	Hexadecanoic acid, methyl ester (Palmitic acid, methyl ester)	15.229
296	C_19_H_36_O_2_	39.9	11-Octadecenoic acid, methyl ester	16.580
298	C_19_H_38_O_2_	16.2	Octadecanoic acid, methyl ester (Stearic acid, methyl ester)	16.760
282	C_18_H_34_O	1.28	Cyclopropaneoctanoic acid, 2-hexyl-, methyl ester	18.706
284	C_18_H_36_O_2_	1.26	Hexadecanoic acid, 15-methyl-, methyl ester	19.985

### Evaluation of collagen-NPs biosynthesis by *Streptomyces xinghaiensis* NEAA-1 cell free supernatant

**Figure 5:**
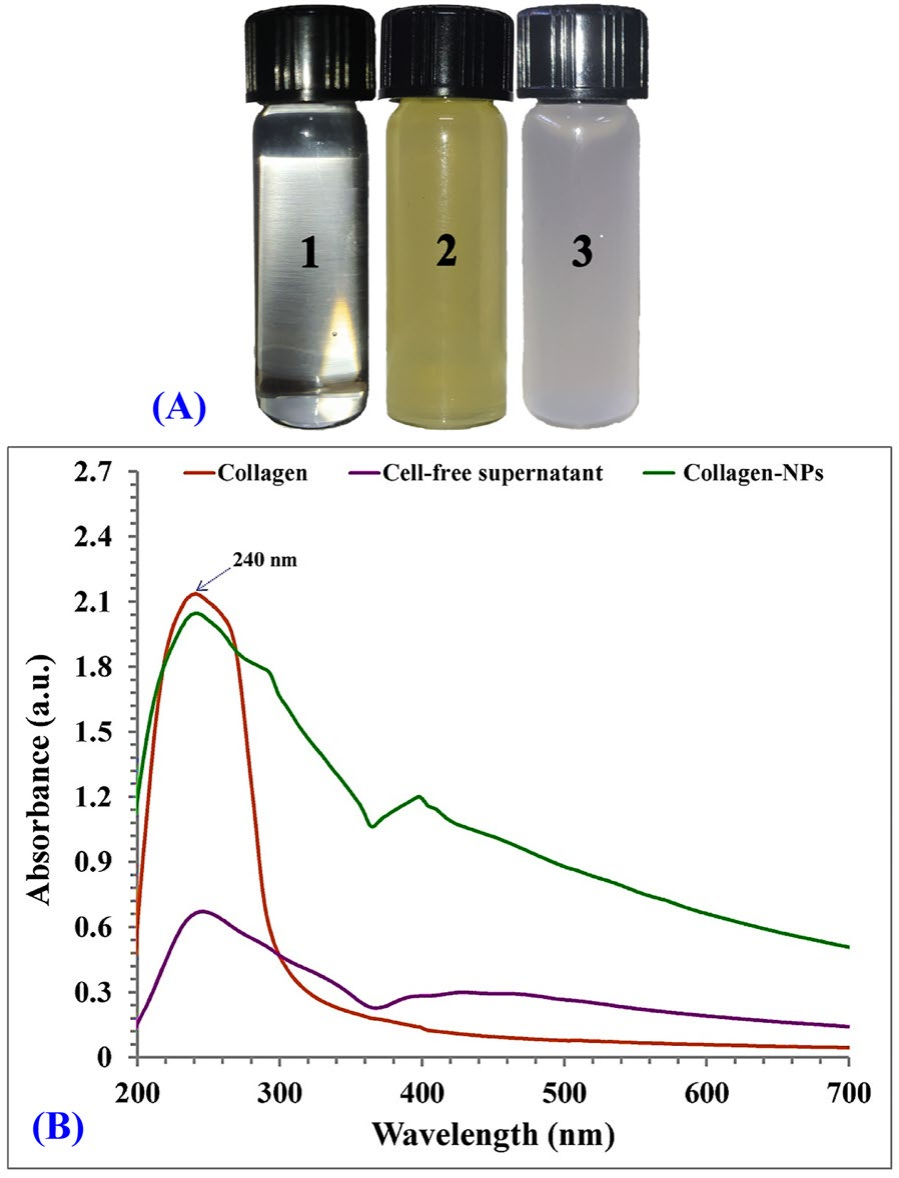
(**A**) Optical observation of collagen soln. (1) Cell-free supernatant of *Streptomyces xinghaiensis* NEAA-1 (2), collagen-NPs (3) & (**B**) UV–visible absorbance of collagen-NPs biosynthesis by *Streptomyces xinghaiensis* NEAA-1.

For the biosynthesis of collagen-NPs (Fig. 5A), 1 mL of 10 mg/mL of pure marine collagen type I solution was added to 9 mL of freshly prepared cell-free supernatant. One of methods of protein NPs fabrication is desolvation. In desolvation method, according to protein concentration, temperature, desolvation factor addition (like alcohol or natural salt) and pH of the medium, the size of protein NPs can be altered^10^. So according to the GC-mass analysis, three types of alcohols (2-methyl-1-hexadecanol, 2-isopropyl-5-methyl-1-hexanol and heptacosanol) can act as desolvation factor and have the ability to fabricate collagen-NPs. Also, aldehyde (5-octadecenal) can act as desolvation factor and form collagen-NPs. The desolvation factor can change the structure of collagen and decreases its solubility such as nanoparticle formation after glutaraldehyde addition to collagen mass^40^. The fatty acids, alcohols and aldehyde are assumed to alter interactions between the collagen molecules for formation collagen-NPs. Almost all the fatty acids existed in the secondary metabolites has antioxidant properties which mean that they have a reduction power to fabricate collagen NPs from collagen protein. Also, the fatty acids are similar to acetic acid as they consist of carboxylic acid with an aliphatic chain except the numbers of carbon atoms. Acetic acid was previously used for fabrication of collagen nanoparticles according to Nagaraja et al*.*^41^ study. Oleic acid is the most attractive fatty acids which previously used in formation of metal nanoparticles and also as a perfect capping agent as in the studies of Cınar et al*.*^42^, Gupta et al*.*^43^, Xia et al*.*^44^ and Mourdikoudis et al*.*^45^. Palmitic acid methyl ester was found in the second degree in abundance which also used as super hydrophobic coating material of ZnO nanoparticles reported by Agrawal et al*.*^46^.

### Spectroscopy analysis of collagen-NPs

UV–visible analysis (Fig. 5B) was performed to determine maximum absorption peak for collagen-NPs, collagen powder and *Streptomyces xinghaiensis* NEAA-1 cell-free supernatant. The maximum absorbance peak of collagen powder recorded at 240 nm. Collagen maximum absorbance peak within a range of 210–240 nm because it is mostly made of glycine followed by proline/hydroxyproline^47^, which confirmed the purity of collagen used. The purity of collagen is further supported by absence of absorption peak at around 280 nm, which correspond to aromatic amino acids like phenylalanine and tyrosine^48^. Also, the maximum absorption of collagen-NPs was observed at 240 nm which confirmed the existence of collagen itself.

### Microscopy analysis of collagen-NPs

**Figure 6:**
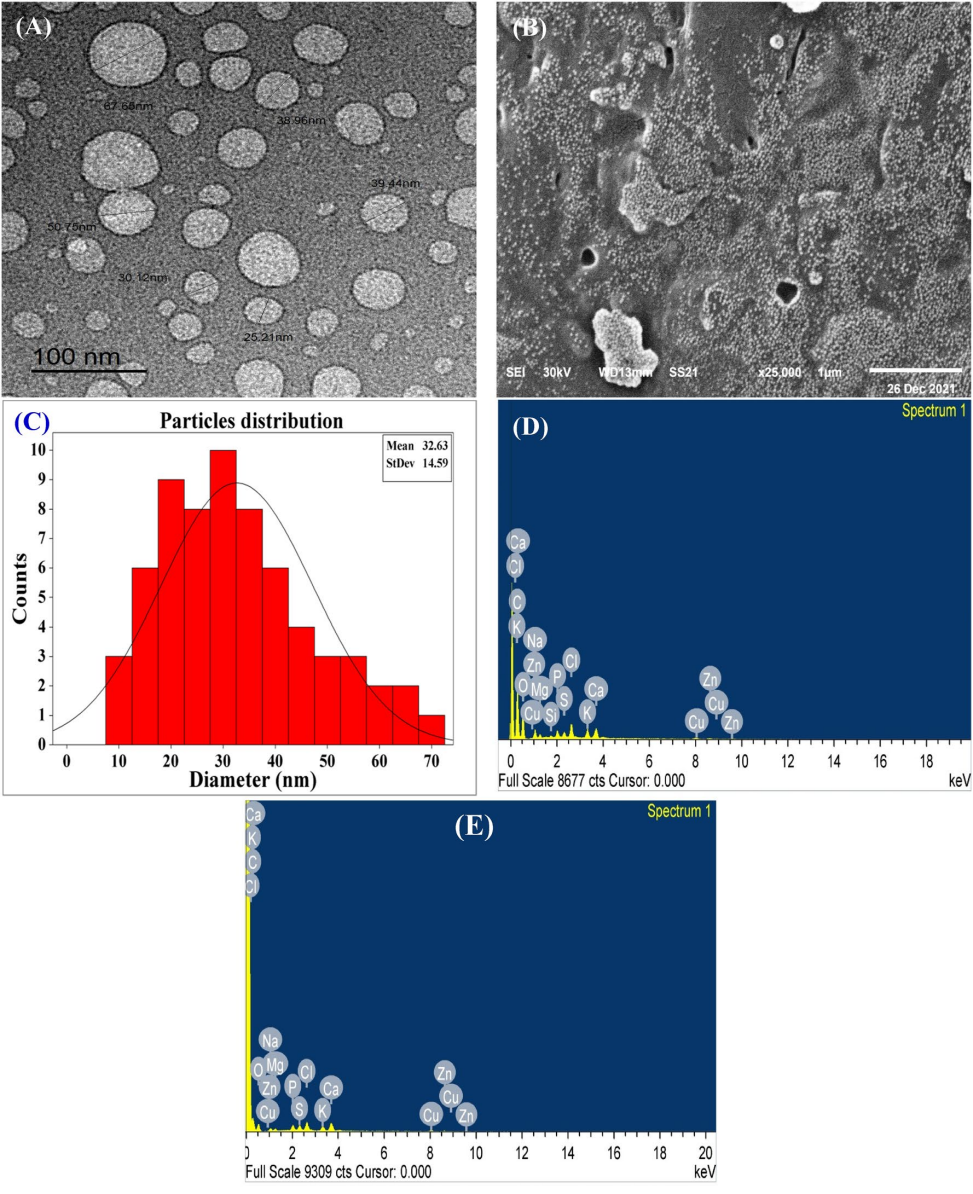
Microscopy investigation of collagen-NPs biosynthesis by *Streptomyces xinghaiensis* NEAA-1: (**A**) TEM image, (**B**) SEM image, (**C**) particles size distribution, EDX of Collagen-NPs and EDX of the cell-free supernatant (**E**).

TEM and SEM are used for investigation and also confirmation of the morphology of collagen-NPs biosynthesized using the cell-free supernatant of *Streptomyces xinghaiensis* NEAA-1 (Fig. 6A,B). TEM showed hollow sphere nanoscale particles with mean of 32.63 nm in diameter and 14.59 of standard deviation (Fig. 6C). The diameter average of our result is considering a perfect result comparing with the average diameter (approximately 100 nm) of Luo et al*.*^49^ who synthesized the collagen-NPs by using a chemical substance and heat. The detailed structural characteristics of NPs sample was evaluated using SEM micrographs^50^.

### Energy-dispersive X-ray (EDX)

The EDX of collagen-NPs powder (Fig. 6D) showed elements peaks for carbon, oxygen, sodium, magnesium, silicon, phosphate, sulfur, chloride, potassium, calcium, copper and zinc. The most abundant element was carbon with weight and atomic percentage 58.73 and 67.12; respectively followed by oxygen with weight and atomic percentage 35.16 and 30.16; respectively. The Cu signal came from the copper grid^51^. In case of Zn, S, P, Cl, Si, Mg, Na, Ca and K, they are elements came from the cell-free supernatant of *Streptomyces xinghaiensis* NEAA-1 (Fig. 6E). Those elements came from the cell-free supernatant media of *Streptomyces xinghaiensis* which presented as trace elements of the media components (starch, MgSO_4_, KNO_3_, NaCl, K_2_HPO_4_, FeSO_4_.7H_2_O and yeast extract). From those trace elements, Ca, Cu, Fe, Pb, Mg, K, S, P, Si and Zn that were presented with NaCl. As (Arsenic), Na, Cl, Pb, NO_3_ and SO_4_ that were presented with K_2_HPO_4_. Yeast extract' trace elements are P, Zn, Fe, K, Co, Mn, Mg, Sr (Strontium) and Cr. Although they were found as traces, gathering them together may lead to the appearance of a signal of them.

### Fourier-transform infra-red (FTIR)

**Figure 7:**
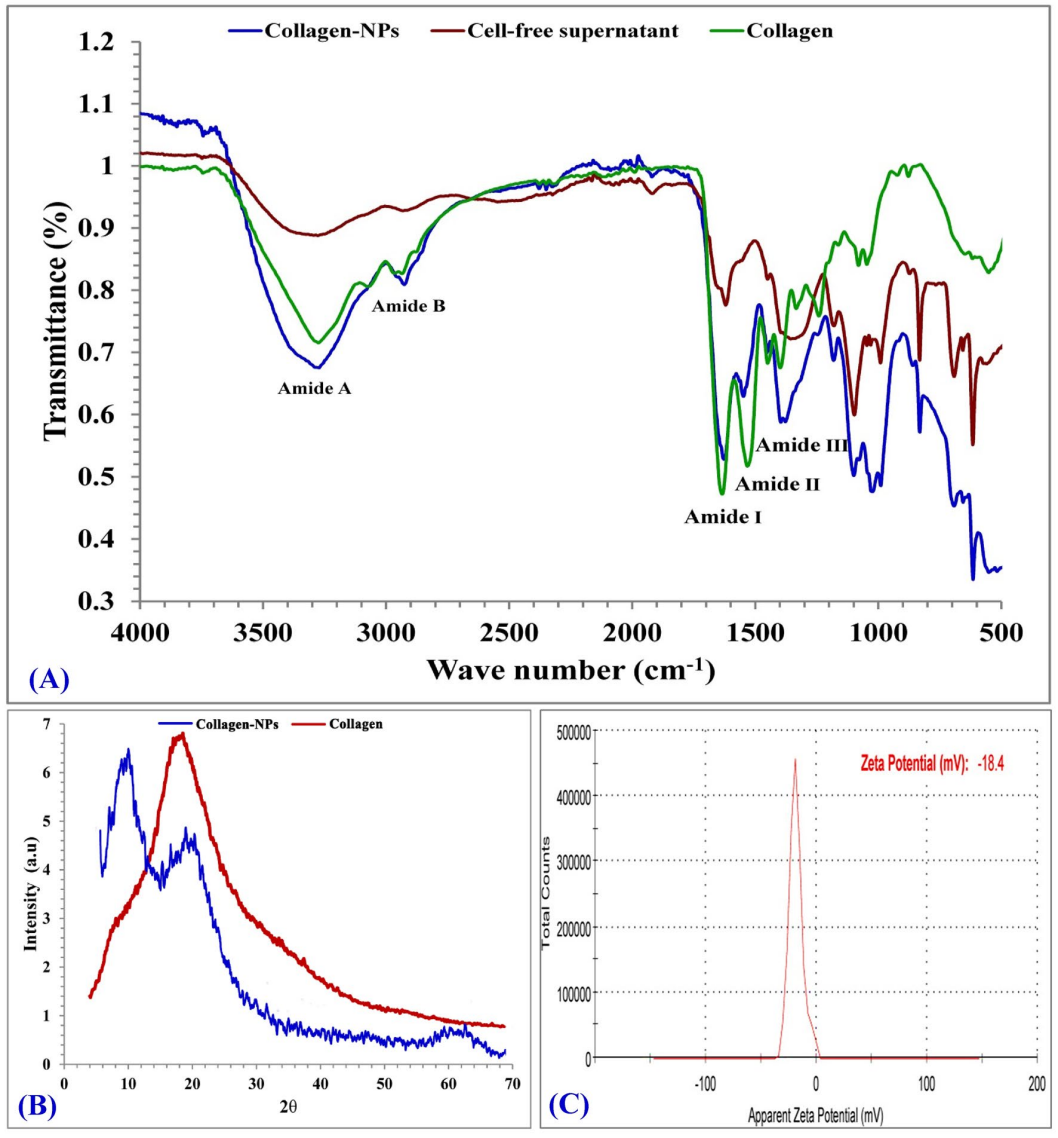
FTIR (**A**), X-ray diffraction analysis (**B**) and Zeta-potential (**C**) of collagen-NPs by biosynthesis by *Streptomyces xinghaiensis* NEAA-1.

FTIR analysis (Fig. 7A) has been long utilized as an effective method for investigating the dynamics and the secondary structures of proteins^52,53^. In FTIR spectrum, showed absorption peaks (3329, 2964, 1655, 1594, 1479, 1403, 1272, 1019, 1110, 1032, 833, 704, 616 cm^−1^) of the obtained collagen nanoparticles. The peak at 3329 cm^−1^ is related to the vibrations of N–H stretching that belong to NH_2_ in aromatic amines, amide A and primary amines that present between 3520 cm^−1^ and 3320 cm^−1^ according to Pati et al*.*^54^ study. Amide B found in the absorption peak at 2964 cm^−1^ found in the range of 2990 to 2850 cm^−1^ and refers to CH of symmetric and anti-symmetric stretching which present in –CH_3_ and –CH_2_– in aliphatic compounds^55^. The characteristic frequencies marker of peptide secondary structure located between 1600 and 1700 cm^−1^ that referred to amide I bond linked with (C=O) group straight with the polypeptide backbone^56^, so in this study was found at 1655 cm^−1^. The peak at 1479 cm^−1^ belongs to NH in secondary amides and present between the normal absorption ranges (1478–1565 cm^−1^) of the amide II^57^. The 1403 cm^1^ peak refers to C–C stretch of aromatic compounds. The absorption peak at 1272 cm^−1^ were found inside the range of 1411 cm^−1^ (amide II) and 1241 cm^−1^ (amide III) band ratio which was nearly equal to 1, that approved the collagen triple helical structure^58^. Peak at 1019 cm^−1^ proved the existence of C–O stretch founded in alcohol and peaks at 1110 and 1032 cm^−1^ proved the existence of C–NH_2_ bond founded in primary aliphatic amines^55^. Peak at 833 cm^−1^ proved the existence of 1,2,4-trisubust benzene, peak at 704 cm^−1^ proved the existence of Ar–OH in phenols and finally peaks at (616 cm^−1^) proved the existence of O–C=O in carboxylic acid^57^.

### X-ray diffraction analysis (XRD)

X-ray diffraction of collagen-NPs and collagen was shown in Fig. 7B. Collagen-NPs exhibit 2 sharp and intense peaks diffractograms at 2*θ* = 10° and 24° which indicates its high crystallinity^59^. This characteristic peaks and broad pattern indicate a typical diffraction pattern for collagen.

### Zeta potential

Marine collagen type I nanoparticles have a narrow range of Zeta potential, which improves catalytic activity, increases dispersion capacity, and increases surface area^60^. According to Fig. 7C, the appropriate Zeta potential value is − 18.4 mV with a standard deviation of 6.24 mV and conductivity of 2.45 mS/cm. The chemically synthesised collagen-NPs have Zeta potential value of − 11.3 mV and − 16.1 mV^61^. Comparing our results to the chemically synthesised collagen-NPs, our green synthesised collagen-NPs using the cell-free supernatant of *Streptomyces xinghaiensis* NEAA-1 showed a great stability than the chemically synthesised collagen-NPs.

### Thermogravimetric analysis (TGA) and Differential scanning calorimetry (DSC)

**Figure 8:**
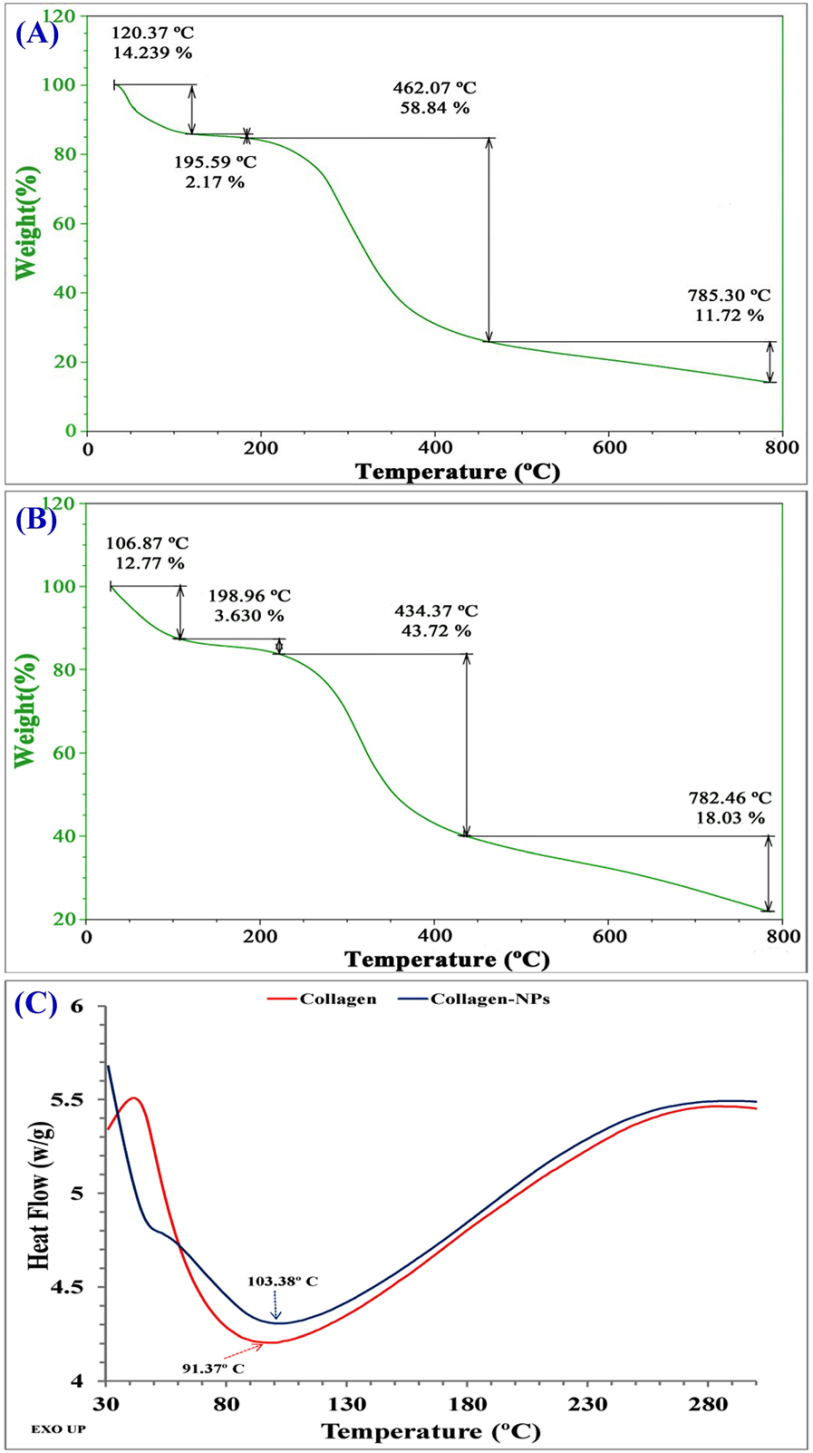
Thermal stability investigation of collagen-NPs: TGA analysis for collagen-NPs (**A**) and collagen (**B**). DSC of collagen-NPs and collagen.

TGA analysis was shown in Fig. 8A. The initial transition step of collagen-NPs is thought to have been triggered by the thermal breakdown of the polypeptide chain of collagen-NPs at 120.37 °C a with weight percentage 14.24. There was a change in the transition temperature and weight percentage when compared to collagen (Fig. 7B) during thermal degradation (106.87 °C and 12.77%, respectively). The initial weight loss is due to the existence of moisture and the evaporation of physiosorbed water according to the study of Iafisco et al*.*^62^. The second transition of collagen was at 198.96 °C and a weight percentage of 3.63, while with collagen-NPs, the second transition was at 195.59 °C and a weight percentage of 2.17. The second decomposition stage is due to the collagen decomposition and consumption of the residue of the collagen organic matrix^63^. The third thermal denaturation of collagen and collagen-NPs is considered the largest transition temperature for both. The weight percentages of collagen and collagen-NPs were 43.72 and 58.84; respectively, and the transition temperature of collagen and collagen-NPs (434.37 and 462.07 °C; respectively). The complete thermal denaturation takes place at the fourth temperature transition, which revealed an elevation in degradation temperature for collagen-NPs compared with collagen. The percentage of final weight left was 18.03 and 11.72; respectively. The final temperature transition was 782.46 °C and 785.3 °C; respectively. The data indicated that collagen-NPs required relatively higher thermal stability due to the presence of bioactive compounds surrounding the nanoparticles.

DSC was first applied to the study of protein denaturation^64^ and it has since provided unique insights into the basic thermodynamics of protein denaturation and protein stability as a result of rigorous handling practises and extended storage conditions^65^. DSC of collagen-NPs (Fig. 7C) showed an endothermic peak at 103.38 °C which was higher than 91.37 °C for collagen, attributed to collagen-NPs and collagen due to helix denaturation^66^. The high endothermic temperature peak of collagen-NPs attributed to the bioactive compounds of the capping agents surround the collagen-NPs comes from the cell-free supernatant of *Streptomyces xinghaiensis* NEAA-1.

### Response surface approach for optimization of collagen-NPs biosynthesis

For optimization of collagen-NPs biosynthesis, 30 different trials with 3 levels (− 1, 0, 1) of different 4 variables, including pH level coded X_1_, temperature coded X_2_, incubation time coded X_3_ and collagen concentration coded X_4_ were performed and represented in Table 3. The highest biosynthesis of collagen nanoparticles was observed in run order 23. The maximum biosynthesis was (8.92 mg/mL) which was achieved in run order 23 under the conditions of 10 mg/mL of collagen concentration, initial pH 7, an incubation time 48 h at 35 °C. However, the minimum biosynthesis was 1.21 mg/mL which was achieved in run order 1 under the conditions of (5 mg/mL) of collagen concentration, initial pH 9, an incubation time 72 h at 45 °C. Predicted and experimented values also represented in Table 3. After FCCD optimization, the yield of collagen-NPs obtained (8.92 mg/mL) was 3.32-fold compared to the yield obtained under non-optimized conditions (2.5 mg/mL).

**Table 3:** Face-centered central composite design matrix representing collagen-NPs biosynthesis by *Streptomyces xinghaiensis* NEAA-1 as affected by pH, temperature, collagen concentrations and incubation time with factor levels (coded and actual).

Std	Run	Variables				Collagen-NPs biosynthesis (mg/mL)		Residuals
		X_1_	X_2_	X_3_	X_4_	Actual	Predicted	
8	1	1	1	1	− 1	1.21	1.09	0.12
9	2	− 1	− 1	− 1	1	6.14	6.08	0.06
3	3	− 1	1	− 1	− 1	2.98	2.92	0.06
21	4	0	0	− 1	0	8.11	8.46	− 0.35
18	5	1	0	0	0	7.25	7.28	− 0.03
11	6	− 1	1	− 1	1	6.00	5.95	0.05
13	7	− 1	− 1	1	1	7.10	7.13	− 0.03
2	8	1	− 1	− 1	− 1	2.34	2.42	− 0.08
14	9	1	− 1	1	1	5.24	5.12	0.12
20	10	0	1	0	0	7.03	7.33	− 0.30
12	11	1	1	− 1	1	5.69	5.50	0.19
17	12	− 1	0	0	0	8.10	8.40	− 0.30
28	13	0	0	0	0	8.87	8.55	0.32
23	14	0	0	0	− 1	4.68	4.46	0.22
7	15	− 1	1	1	− 1	3.01	3.16	− 0.15
6	16	1	− 1	1	− 1	2.01	2.16	− 0.15
4	17	1	1	− 1	− 1	2.62	2.69	− 0.07
15	18	− 1	1	1	1	5.92	5.67	0.25
5	19	− 1	− 1	1	− 1	3.93	3.94	− 0.01
30	20	0	0	0	0	8.71	8.55	0.16
29	21	0	0	0	0	8.82	8.55	0.27
22	22	0	0	1	0	8.20	8.19	0.01
27	23	0	0	0	0	8.92	8.55	0.37
10	24	1	− 1	− 1	1	5.97	5.91	0.06
24	25	0	0	0	1	6.91	7.46	− 0.55
25	26	0	0	0	0	8.59	8.55	0.04
19	27	0	− 1	0	0	7.88	7.92	− 0.04
16	28	1	1	1	1	3.22	3.38	− 0.16
26	29	0	0	0	0	8.42	8.55	− 0.13
1	30	− 1	− 1	− 1	− 1	2.43	2.37	0.06

Variable (g/L)	Code	Coded and actual levels						
		− 1	0	1				
pH	X_1_	5	7	9				
Temperature (°C)	X_2_	25	35	45				
Incubation time (h)	X_3_	24	48	72				
Collagen concentration (mg/mL)	X_4_	5	10	15				

### Multiple regression analysis and ANOVA

The relationship between the independent variables and their interactions on collagen-NPs biosynthesis is explained by the second order polynomial model. According to the study of El-Naggar et al*.*^67,68^, when the R^2^-value is more than 0.9, there will be a strong connection between the predicted and actual values of the response. Because of this, the current R^2^-value (0.993) demonstrated a very strong fit between the actual and predicted values, suggesting that the model is trustworthy for the biosynthesis of collagen-NPs in the current investigation. Additionally, the very high adjusted determination coefficient (Adj. R^2^ = 0.9864) indicates that the model has a high level of relevance^69,70^. The predicted (Pred. R^2^) value is 0.9722 which has a satisfactory adjustment between the actual and predicted values of collagen-NPs, as well as a reasonable agreement with the adjusted R^2^. Collagen-NPs biosynthesis statistical analysis exhibits 4.88% of coefficient of variation percentage (C.V. %), which is comparatively very low and indicates great accuracy, dependability, and precision of experimental trials^71^.

The model's Adequate Precision recorded was 36.81 with a mean of 5.88 and a standard deviation of 0.29 (Table 4). Adequate precision assesses the signal-to-noise ratio, a ratio greater than four indicates that the model has high accuracy and appropriation of the design^72^. The adequate precision of 36.81 indicates the high accuracy and appropriation of the design for optimizing collagen-NPs biosynthesis at different levels of the evaluated variables.

**Table 4:** Variance analysis for collagen-NPs biosynthesis by *Streptomyces xinghaiensis* NEAA-1 as affected by pH, incubation time, collagen concentrations and temperature with factor levels (coded and actual).

Source of variance		Coefficient estimate	Degrees of Freedom	Sum of Squares	Mean Square	*F*-value	*P*-value
Model		8.55	14	173.78	12.41	151.16	< 0.0001*
Linear effect	X_1_	− 0.56	1	5.62	5.62	68.47	< 0.0001*
	X_2_	− 0.30	1	1.60	1.60	19.44	0.0005*
	X_3_	− 0.14	1	0.33	0.33	4.03	0.0631
	X_4_	1.50	1	40.44	40.44	492.47	< 0.0001*
Interaction effect	X_1_X_2_	− 0.07	1	0.08	0.08	0.97	0.3399
	X_1_X_3_	− 0.46	1	3.38	3.38	41.12	< 0.0001*
	X_1_X_4_	− 0.05	1	0.05	0.05	0.58	0.4596
	X_2_X_3_	− 0.33	1	1.78	1.78	21.62	0.0003*
	X_2_X_4_	− 0.17	1	0.47	0.47	5.67	0.0309*
	X_3_X_4_	− 0.13	1	0.28	0.28	3.39	0.0855
Quadratic effect	X_1_^2^	− 0.71	1	1.31	1.31	15.92	0.0012*
	X_2_^2^	− 0.93	1	2.24	2.24	27.31	0.0001*
	X_3_^2^	− 0.23	1	0.14	0.14	1.67	0.2153
	X_4_^2^	− 2.59	1	17.38	17.38	211.71	< 0.0001*
Error effect	Lack of Fit		10	1.05	0.11	2.93	0.1234
	Pure Error		5	0.18	0.04		
R^2^	0.993	Std. Dev	0.29				
Adj R^2^	0.9864	Mean	5.88				
Pred R^2^	0.9722	C.V. %	4.88				
Adeq Precision	36.81						

*C.V: Coefficient of variation, *P*: Level of significance, *F*: Fishers's function.

Table 4 displays the ANOVA results for the experimental design, the Fisher (*F*-value), the probability (*P*-value), the sum, and the mean squares. The model's *F*-value is (151.16) and its *P*-value is less than 0.0001, making it a model that is thought to be very appropriate. To assess each coefficient's significance and determine the pattern of the test variables' mutual interaction, the probability value of each coefficient was determined. Coefficients with smaller *P-*values (less than 0.05) are more significant^69,73^. With a *P*-value of less than 0.0001, the linear impacts of X_1_ (pH), and X_4_ (collagen concentration) and a *P*-value of 0.0005 for X_2_ (temperature) demonstrated the maximum significance of these variables.

The coefficient estimates also indicated if there were favorable or unfavorable effects on collagen-NPs. Positive or negative, a large estimated effect suggests that the independent variables have a significant impact on the response. Any tested variable whose expected effect shows a positive sign indicates that production grows at high levels of the variable. If the sign is negative, it implies that production increases at low levels of the variable. There are two interactions between two variables: antagonism (negative coefficient) and synergism (positive coefficient)^74^. The positive coefficient shown for X_4_ only suggests that this variable boosts the biosynthesis of collagen-NPs in a linear way, while the rest of the coefficients are negative. The biosynthesis of collagen nanoparticles increases as the collagen concentration increases. While the linear effects of other parameters and both interaction and quadric effects of all parameters have negative coefficients (Table 4).

A higher *P*-value indicates that the presented data show that the model's lack-of-fit error did not reach the significance threshold. The interaction effects of X_1_ (pH) with X_3_ (incubation time) and X_2_ (temperature) with X_3_ (incubation time) on the biosynthesis of collagen-NPs were the most significant. The quadric effect of X_1_^2^ (pH), X_2_^2^ (temperature) and X_4_^2^ (collagen concentration) also showed maximum significance on collagen nanoparticles biosynthesis. However, the interaction effect between X_1_ (pH) and X_4_ (collagen concentration) and the quadric effect of X_3_^2^ (incubation time) didn't show any significant effect on collagen nanoparticles biosynthesis. The fit summary results provided in Table 5 were used to choose between the 2FI, linear and quadratic models as the most suitable polynomial model for collagen-NPs biosynthesis by *Streptomyces xinghaiensis* NEAA-1. With higher values of the adj. R^2^ (0.9864) and predicted R^2^ (0.9722), the quadratic model is the suitable and suggested model for collagen-NPs biosynthesis, where lack of fit (*P*-value = 0.1234; *F*-value = 2.93) is non-significant.

**Table 5:** Fit summary for face-centered central composite design results for collagen-NPs biosynthesis by *Streptomyces xinghaiensis* NEAA-1 as affected by pH, incubation time, collagen concentrations and temperature with factor levels (coded and actual).

Lack of fit tests					
Source	Sum of squares	*Df*	Mean square	*F-*value	*P-*value *P*rob > *F*
Linear	126.84	20	6.34	176.68	< 0.0001
2FI	120.82	14	8.63	240.41	< 0.0001
Quadratic	1.05	10	0.1052	2.93	0.1234

Fit summary					
Source	Sequential *P-*value	Lack of fit *P-*value	Adjusted R^2^	Predicted R^2^	
Linear	0.0806	< 0.0001	0.1581	− 0.0288	
2FI	0.9850	< 0.0001	− 0.0553	− 1.5438	
Quadratic	< 0.0001	0.1234	0.9864	0.9722	Suggested

Model summary statistics					
Source	Standard deviation	R-squared	Adjusted R-squared	Predicted R-squared	PRESS
Linear	2.25	0.2742	0.1581	− 0.0288	180.05
2FI	2.52	0.3086	− 0.0553	− 1.5438	445.2
Quadratic	0.2866	0.993	0.9864	0.9722	4.86

*PRESS: sum of squares of prediction error, 2FI: two factors interaction, *df*: degree of freedom.

The second order polynomial model (Eq. 2) has been used for the determination the maximum collagen-NPs that correspond to the four variables optimum levels and the evaluation of the relationship between the dependent variable and independent variables; pH, temperature, incubation time, and collagen concentration.

$$\begin{aligned} {\text{Y}} & = {8}.{55} - 0.{\text{56 X}}_{{1}} - 0.{\text{3 X}}_{{2}} - 0.{\text{14 X}}_{{3}} + {1}.{\text{5 X}}_{{4}} - 0.0{\text{7 X}}_{{1}} {\text{X}}_{{2}} \\ & \quad - 0.{\text{46 X}}_{{1}} {\text{X}}_{{3}} - 0.0{\text{5 X}}_{{1}} {\text{X}}_{{4}} - 0.{\text{33 X}}_{{1}} {\text{X}}_{{3}} - 0.{\text{77 X}}_{{2}} {\text{X}}_{{4}} - 0.{\text{13 X}}_{{3}} {\text{X}}_{{4}} \\ & \quad - 0.{\text{71 X}}_{{1}}^{{2}} - 0.{\text{93 X}}_{{2}}^{{2}} - 0.{\text{23 X}}_{{3}}^{{2}} - {2}.{\text{59 X}}_{{4}}^{{2}} \\ \end{aligned}$$

### Model significance check

**Figure 9:**
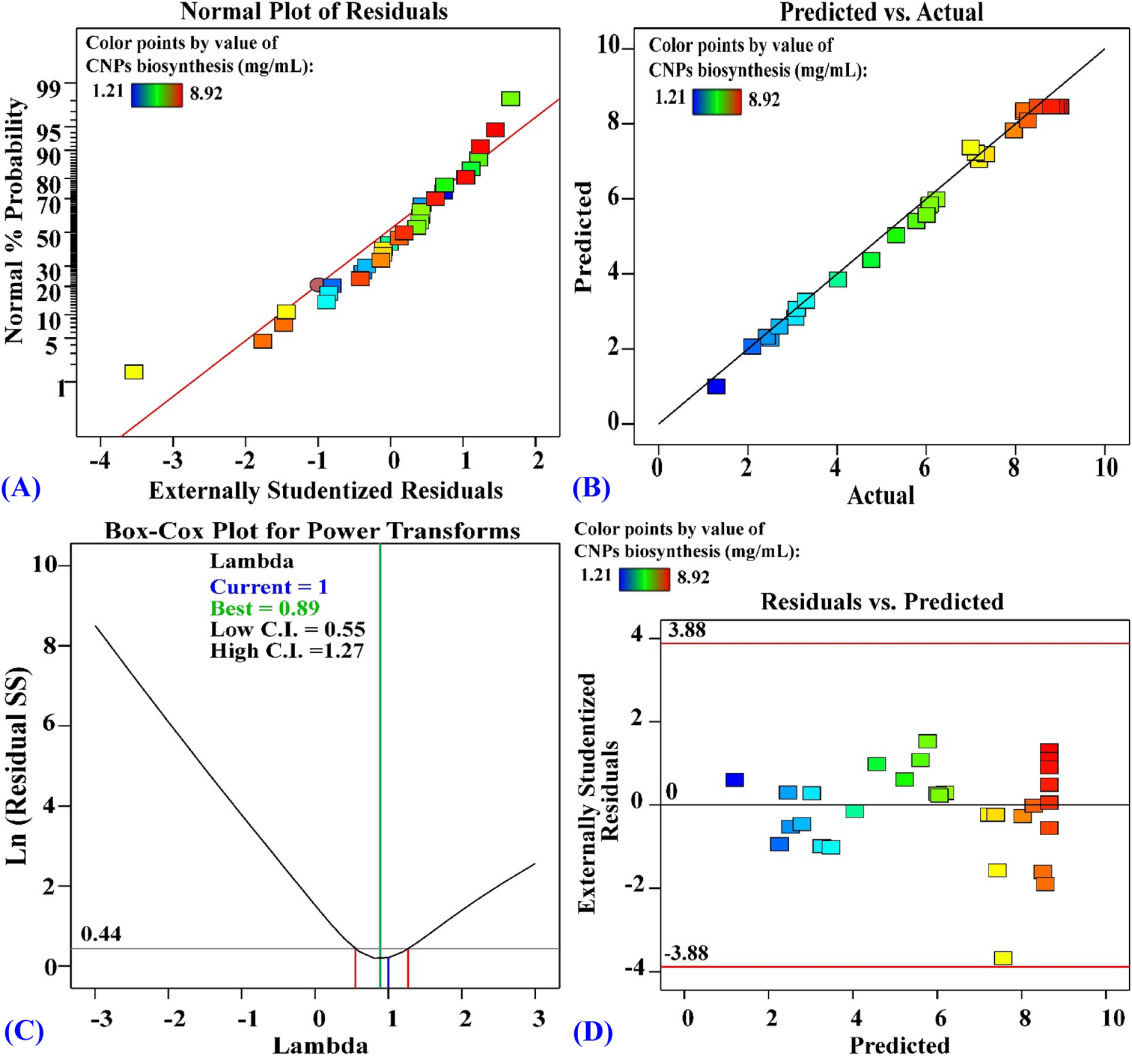
(**A**) Normal probability plotting of internally studentized residuals, (**B**) plotting of actual versus predicted (**C**) Box–Cox plotting of model transformation and (**D**) plotting of internally studentized residuals versus predicted values of collagen-NPs biosynthesis by *Streptomyces xinghaiensis* NEAA-1. *CNPs* collagen-NPs.

The fitted model is checked with the normal probability plot, a substantial diagnostic tool, to reveal and detect the systematic departures from normality^75^, improve a sufficient approximation to the real system, and prevent misleading findings. According to the study of Samuel & Oladipupo^76^, the difference between theoretical and experimental data is defined as residual, and a small residual indicates considerable model accuracy. Figure 9A, displays the residuals normal probability plot, which needed for detection and explanation the systemic departures for the assumption. Externally studentized residuals were plotted against the normal percentage probability, showing that the dot are near to the diagnostic line, so the error variance s homogeneous and the errors are normally distributed and indicating a good model fit^77^. This demonstrated how well the model matched the results of the experiment. Figure 9B displays a plot between the predicted and the biosynthesized collagen-NPs actual values. The points aligned around the diagnostic line, suggesting that there is satisfactory correlation and close agreement between the experimented values and predicted values that confirming the model adequacy^78,79^. Figure 9C displays the power transformation graph (Box–Cox plot). The current λ transformation (λ = 1) is represented in the blue line and the λ value (λ = 0.89) is represented in the green line, while the values of 95% confidence interval, minimum and maximum, are 0.55–1.27; respectively, represented in the red lines. The blue lines of the model localized in the optimal zone inside the two red lines of the maximum and minimum confidence intervals values. Also, the model didn't need any transformation as the current interval (λ = 1) was extremely close to the best value for the model's parameter (λ _best_ = 0.89), demonstrating that the model adequately fits the analysis of variance's underlying premise. Figure 9D plots the residuals against the predicted values. The distance from line 0 represents how that value was predicted to be. On the X-axis, residuals with positive values indicate that the prediction was too high, and those with negative values indicate that it was too low. Line 0 indicates that the guess was 100% accurate. The plot's residual points almost all lined up near the 0 line with no distinct pattern, showing that the experimental and predicted values are fairly close to one another and a good fit of the model^80,81^.

### Three-dimensional plot

**Figure 10:**
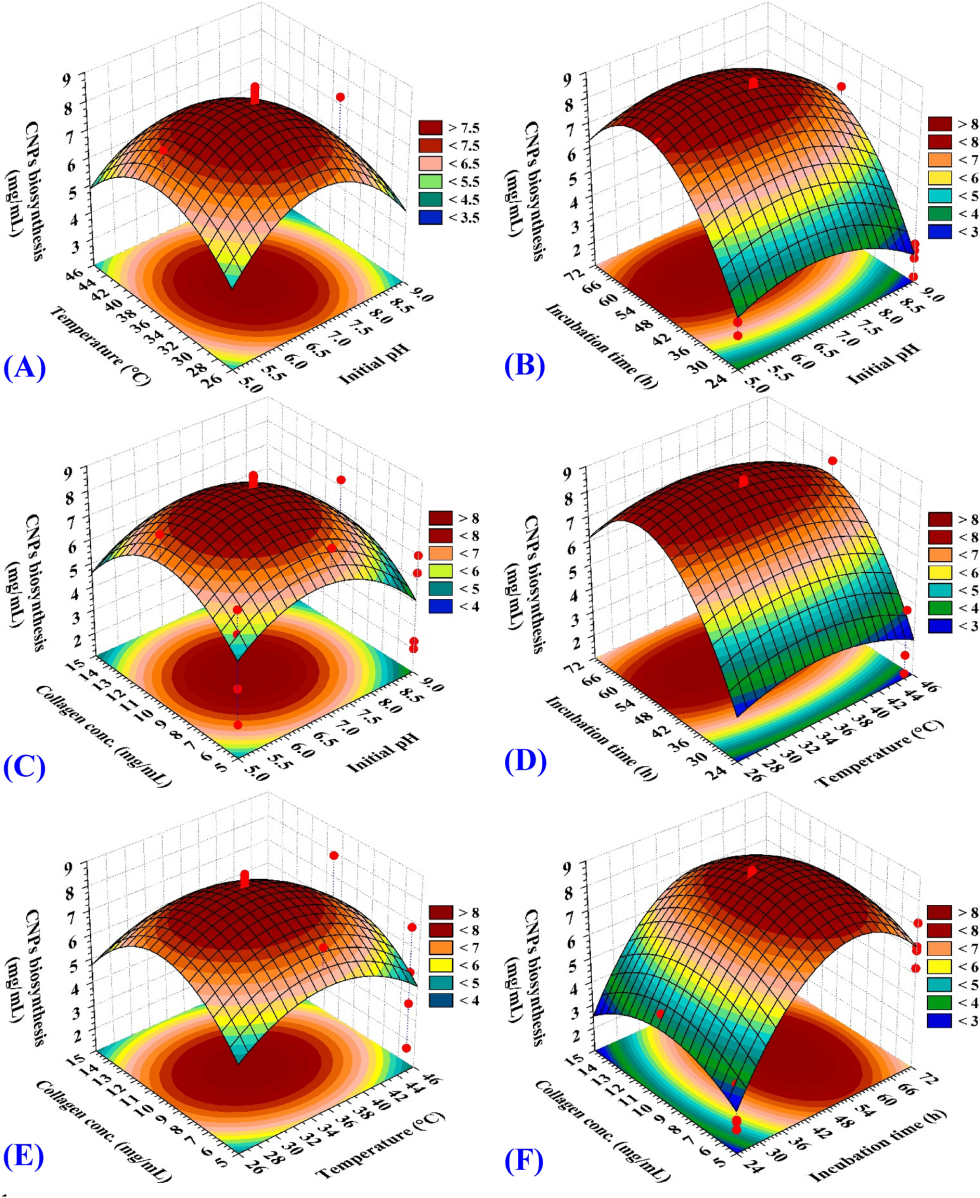
3D plots showing the mutual effects of pH (X_1_), temperature (X_2_), incubation time (X_3_) and Collagen concentration on collagen nanoparticles biosynthesis by *Streptomyces xinghaiensis* NEAA-1. *CNPs* collagen-NPs.

The interaction effects between pairwise combinations of the four independent bioprocess variables on collagen-NPs biosynthesis and their optimal levels were determined by plotting the three-dimensional plots as illustrated in Fig. 10A–F. Two of the independent variables are kept at their zero level and the other two variables are allowed to vary. Collagen-NPs are employed on the Z-axis in opposition to the two independent variables. The type or the concentration of collagen, temperature, pH, and ionic strength are said to have an impact on the in vitro synthesis of collagen nanostructures^82^. Figure 10A displays X_1_ (initial pH) and X_2_ (temperature) effects, whereas X_3_ (incubation time) and X_4_ (collagen concentration) had been maintained at zero levels (48 h and 10 mg/mL; respectively). By progressively raising the temperature and initial pH to the middle point (neutral), the collagen-NPs steadily increase. After that, when pH and temperature rise, collagen-NPs will diminish. Song et al*.*^83^ reported that ionic bonding and hydrogen bonding between collagen molecules were collapsed by excessive acid or alkali. The most significant influencing factor is the pH of the media, which may change the collagen molecules charge distribution and their electrostatic interaction, which alter their ability to form nanostructures^84^. Many researches utilized pH ranged from 7 to 7.4 for collagen nanostructures formation. The charge of collagen amino acids is balanced near the isoelectric point (pH 7.2 for collagen type I); therefore, the collagen molecules favor nanostructures formation^85^. Figure 10B displays X_1_ (initial pH) and X_3_ (incubation time) effects, whereas X_2_ (temperature) and X_4_ (collagen concentration) had been maintained at zero levels (35 °C and 10 mg/mL; respectively). Collagen-NPs biosynthesis increased rapidly with the increase of pH until reaching a specific point then decreased rapidly with the high level of pH. Figure 10C displays X_1_ (pH) and X_4_ (collagen concentration) effects, whereas X_2_ (temperature) and X_3_ (incubation time) had been maintained at zero levels (35 °C and 48 h; respectively). The collagen-NPs increase gradually with the increase of collagen concentration and initial pH, then decrease gradually with the increase of both of them. Figure 10D displays X_2_ (temperature) and X_3_ (incubation time) effects, whereas X_1_ (initial pH) and X_4_ (collagen concentration) had been maintained at zero levels (7 pH and 10 mg/mL; respectively). The collagen-NPs steadily rise by gradually increasing the temperature and incubation time until they reach the intermediate point (neutral). Collagen-NPs will then decrease as incubation time and temperature increase. Figure 10E displays X_2_ (temperature) and X_4_ (collagen concentration) effects, whereas X_1_ (initial pH) and X_3_ (incubation time) had been maintained at zero levels (7 pH and 48 h; respectively). As temperature and collagen concentration got higher, collagen-NPs production gradually increased until it reached the midpoint, then decreased gradually with the excess of both of them due to the high concentration of collagen and inability of the cell-free filtrate to reduce the collagen protein. The most favorable temperature for collagen-NPs formation was room temperature ranged between 35 and 37 °C. Luo et al*.*^49^ was reached to form a well-defined nano-vesicles with diameters of approximately 100 nm have been formed from collagen-like peptide with a transition temperature of 37 °C. Figure 10F displays X_3_ (incubation time) and X_4_ (collagen concentration) effects, whereas the X_1_ (initial pH) and X_2_ (temperature) had been maintained at zero levels (7 and 35 °C; respectively). Collagen-NPs biosynthesis increased rapidly with the increase of collagen concentration and the incubation time until reached at a specific limit, then due to the high concentration of collagen and inability of the cell-free supernatant to reduce the collagen, the biosynthesis decreased rapidly. Also, the long incubation time affected on the biosynthesis and made nanoparticles aggregation.

**Figure 11:**
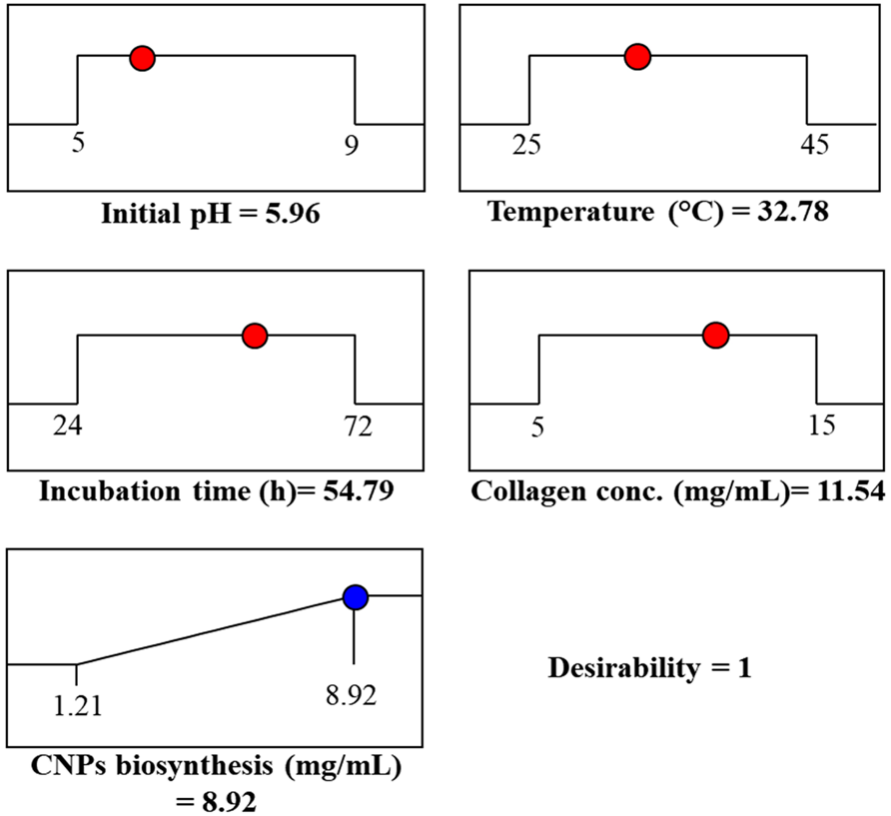
Optimization plotting reveals the optimum predicted values for maximum collagen-NPs biosynthesis by *Streptomyces xinghaiensis* NEAA-1 and the desirability value. *CNPs* collagen-NPs.

Desirability function analysis (DFA) is one of the most widely approaches used for multi-factors optimization to determine the optimal prediction conditions for maximizing the responses^86^. The desirability functions values lie between 0 and 1. The value 1 is refers that the factors give their optimal performance while the value 0 refers to undesirable performance. In Fig. 11, DFA has been done by Design Expert Software (Version 7). Desirability function of the optimum predicted condition reached to 1 for the maximum collagen-NPs biosynthesis (8.92 mg/mL) with 5.96 pH, 32.78 °C of temperature, 54.79 h of incubation time and 11.54 mg/mL of collagen concentration. The results of DFA prediction are almost near the experimental results with (8.71 mg/mL), indicating that there a perfect adjustment between them.

### Anti-hemolytic activity of collagen-NPs

Erythrocytes cell wall contains high concentrations of hemoglobin, oxygen and high poly-unsaturated fatty acid which are oversensitive to the oxidative disruption induced by free radicals resulted in hemolysis and release of haemoglobin into the surrounding blood plasma^87^. Free radicals can attack the lipids membrane resulted in converting the lipids to lipid hydroperoxides which reverse the structure and function of cell wall^88^. In this research, a hydrophilic azo-compound (AAPH) has been used to create 2-amidinopropyl radicals which also named C-radicals within a procedure with particular temperature. Therefore, oxygen molecules can react with C-radicals for generating peroxyl radicals and by increasing time exposure of erythrocyte to AAPH, hemolysis increases^89^.

Collagen-NPs showed anti-hemolythic percentage of 96.1% and standard L-ascorbic acid (vitamin C) has showed anti-hemolytic percentage of 95.8% against the AAPH free radicals induced hemolysis. Anti-hemolytic potency may be due to the action of anti-oxidation power of O‒H group existed in complexes embedded phenol or Ar‒OH or ‒NH_2_ in aromatic amines complexes present in collagen-NPs and confirmed by FTIR. Iwatsuki et al*.*^90^ reported that aromatic amines have a powerful anti-oxidation potency, however amino substituted phenol is considered in more effective than amino-substituted ones.

### In vitro cytotoxicity of collagen-NPs on cancer and normal cell lines

**Figure 12:**
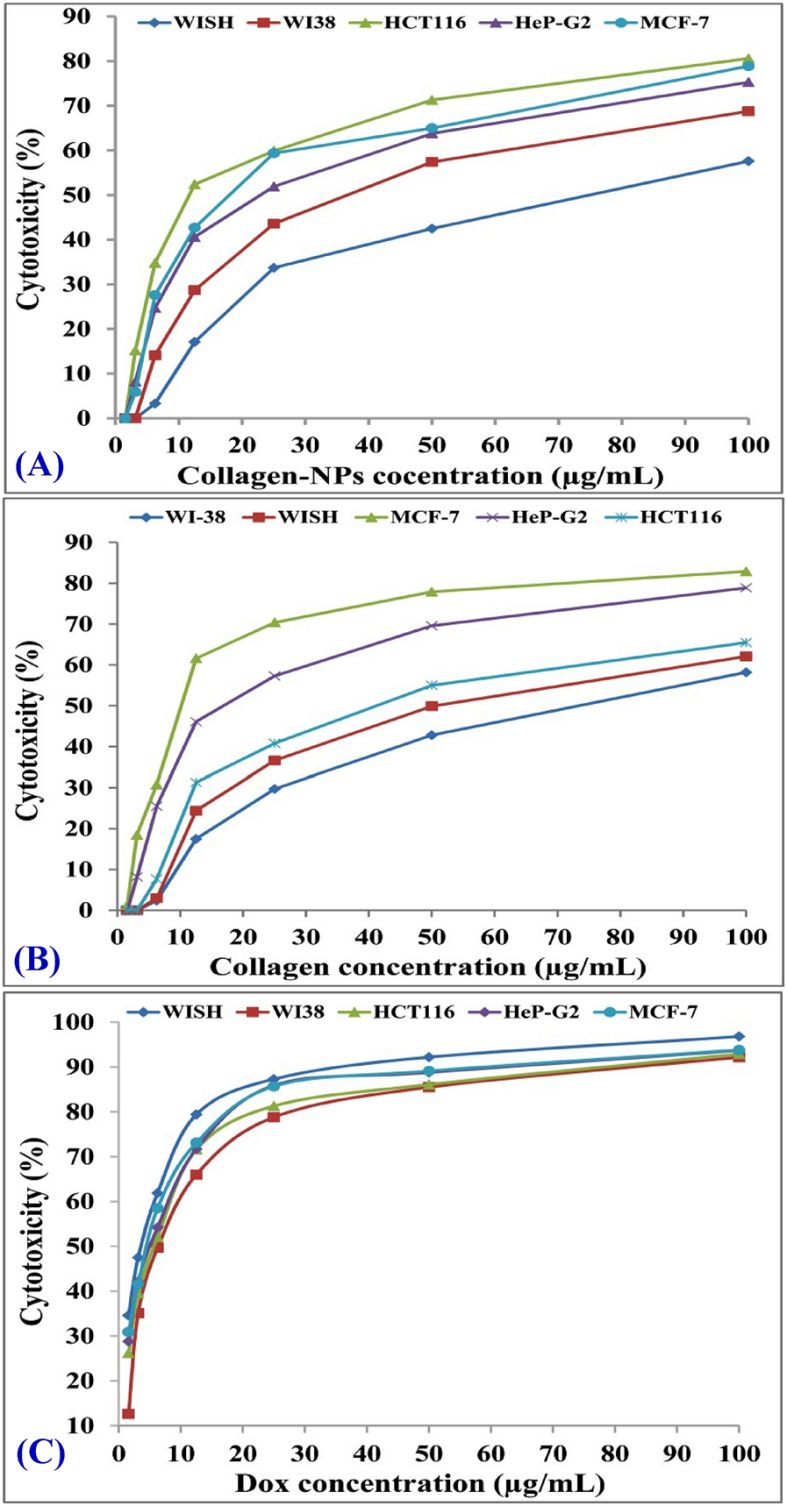
Diagram showing the cytotoxicity effect of collagen-NPs produced using *Streptomyces xinghaiensis* NEAA-1 (**A**) and collagen (**B**) against different normal and cancer cell lines at different concentrations ranged from 1.65 µg to 100 µg. (**C**) Doxorubicin used as standard anticancer drug.

In this study, human lung fibroblast (WI38), human amnion (WISH), human colorectal carcinoma (HCT116), human liver cancer (HeP-G2), and mammary gland breast cancer (MCF-7) cell lines were used to test the cytotoxic effect of collagen-NPs (Fig. 12A) and collagen (Fig. 12B) in vitro. Doxorubicin (DOX), a commercially available anticancer drug, served as the standard (Fig. 12C). With an increase in collagen-NP concentration, the rate of cytotoxicity against cell lines rises. After 48 h of incubation, the inhibitory effect was noticed. Table 6 shows the results as growth inhibitory concentrations (IC_50_) values, which are the chemical concentrations necessary to result in a 50% suppression of cell growth after 48 h of incubation, in comparison to untreated controls. The human colorectal carcinoma (HCT116) was observed at 41.67 ± 2.2 µg/mL (moderate effect), human liver cancer (HeP-G2) was observed at 19.60 ± 1.2 µg/mL (strong effect), and mammary gland breast cancer (MCF-7) was observed at 11.62 ± 0.8 µg/mL (strong effect) for collagen-NPs against cancer cell lines. The results showed a superior anticancer effect of collagen-NPs against two cell lines (HeP-G2 and MCF-7) than collagen alone, while against HCT116, the opposite happened. This behavior could be due to the nature or genetics of the HCT116 cell lines. Cancer cell resistance to anticancer agents can be due to many factors, such as the individual’s genetic differences^91^. Against normal cell lines including human amnion (WISH) cell lines and human lung fibroblast (WI38), however, the IC_50_ of collagen-NPs for cell suppression was found to be 53.25 ± 2.8 and 66.79 ± 3.3 µg/mL, respectively. Therefore, compared to WISH and WI38 normal cell lines, the biosynthesized collagen-NPs had a greater cytotoxic effect on the breast cancer cell lines MCF-7, HeP-G2, and HCT116. A quick colorimetric test for determining cell viability is being considered via MTT assay. This assay measures the amount of mitochondrial succinate dehydrogenase, which converts the yellow soluble dye MTT (3-(4,5-dimethylthiazol-2-yl)-2,5-diphenyltetrazolium bromide) into the insoluble purple formazan product. Therefore, when cells die, they are no longer able to convert MTT into formazan, and the presence of a purple color is a sign that the cells are still alive. Collagen-NPs have a cytotoxic effect on the mitochondrial activity whereas the decrease in MTT-formazan production due to the reduction of the NADH, D-glucose or concentration in the culture medium according to Winikoff et al*.*^92^ study. Not only MTT is reduced by mitochondrial enzymes, but also by the enzyme’s endosomal compartments (endoplasmic reticulum & Golgi apparatus) and lysosomal compartments^93^. It was reported that NPs can deplete glutathione, deplete the generation of ATP, stimulate protein damage and lysosomes disintegrate^94^. Collagen-NPs have a lethal effect on the cancer cells, due to the substantial synthesis (accumulation) of reactive oxygen species (ROS), which are chemically embedded reactive oxygen as hydroxyl radicals (OH^−^), hydrogen peroxide (H_2_O_2_) and reactive superoxide (O_2_^−^). Peroxisomes and the endoplasmic reticulum (ER) and the mitochondria are where ROS are created the most frequently^95^. By interfering with the electron transport mechanism, NPs have also been shown to increase the buildup of ROS within cells^96^ and interfering mitochondrial function^97^, ration elevating between the NADP^+^ and NADPH^98^, interfering expression of oxidative stress-related genes expression, like *ahpC*, *oxyR*, soxR and *soxS*^99^ and the NADPH production-related gene *met9*^98^, causing in cell damage, lactate dehydrogenase (LDH) increasing and lipid peroxidation^100^, causing of chromosome disintegrate, DNA breakage ( even double or single strand) and aneuploid genic events^101,102^.

**Table 6:** Showing the growth inhibitory concentration (IC_50_) values of collagen-NPs produced using *Streptomyces xinghaiensis* NEAA-1, collagen, doxorubicin against different normal and cancer cell lines.

Component	In vitro cytotoxicity IC_50_ (µg)				
	WISH	WI38	HCT116	HeP-G2	MCF-7
DOX	3.34 ± 0.2	6.72 ± 0.5	5.23 ± 0.3	4.50 ± 0.2	4.17 ± 0.2
Collagen	66.03 ± 3.2	37.25 ± 2.4	15.61 ± 1.2	24.48 ± 1.9	20.69 ± 1.5
Collagen-NPs	53.25 ± 2.8	66.79 ± 3.3	41.67 ± 2.2	19.60 ± 1.2	11.62 ± 0.8

*IC_50_ (µg/mL): over 100 (non-cytotoxic), 51–100 (weak), 21–50 (moderate), 11–20 (strong) and 1–10 (very strong); DOX: Doxorubicin.

### In vivo Ehrlich apoptosis stimulation by collagen-NPs

**Figure 13:**
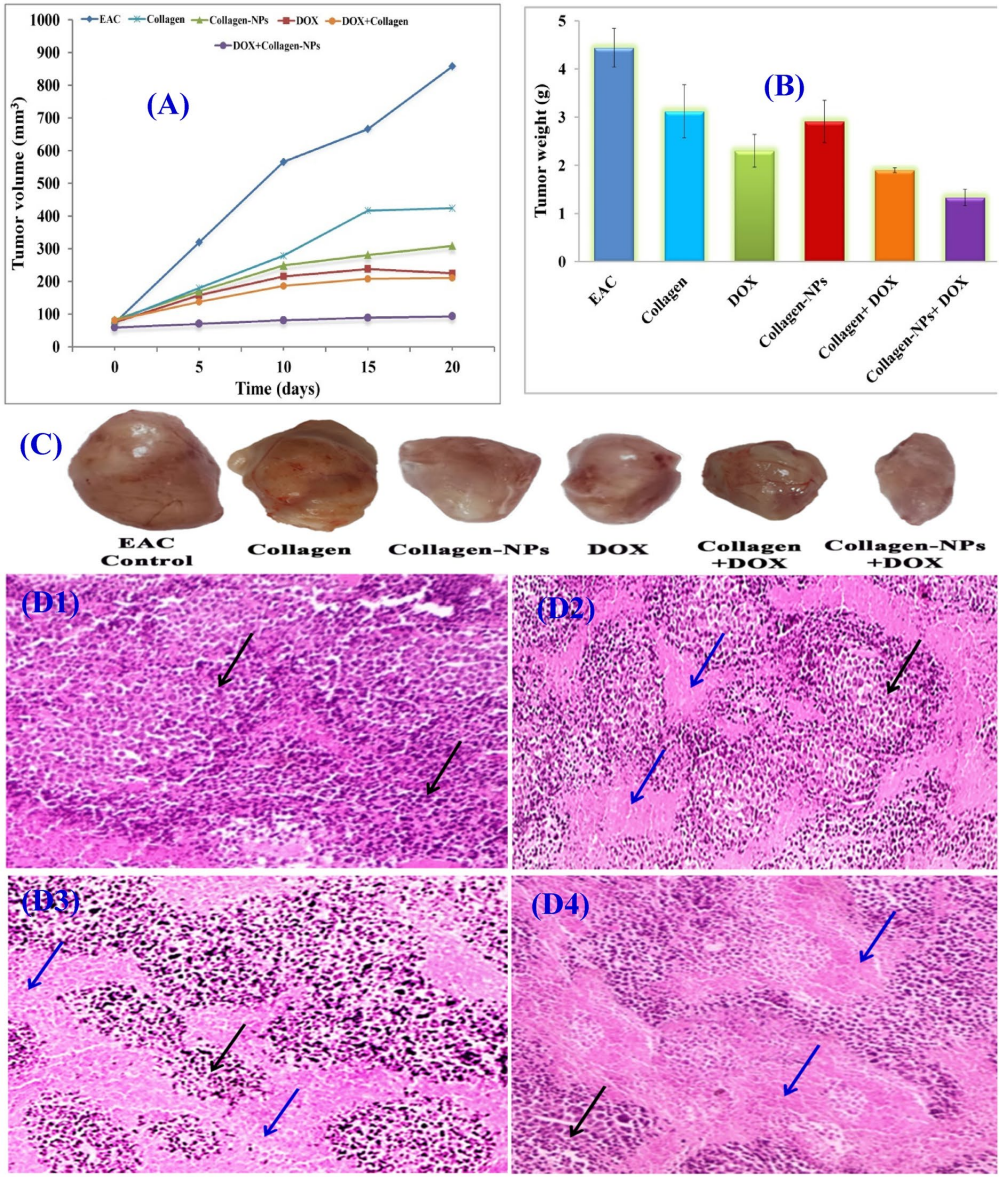
Effect of collagen-NPs by *Streptomyces xinghaiensis* NEAA-1 and collagen curing alone or in integration with DOX on tumor volume (**A**) and tumor weight of EAC bearing mice (**B**); images of solid tumors at the same power of magnification, zooming and distance from camera (**C**); histopathological analysis micrographs on tumor sections of untreated mice bearing EAC (D1), mice bearing EAC cured with collagen-NPs (D2), mice bearing EAC cured with DOX and mice bearing EAC cured with collagen-NPs integrated with DOX (D4); Black arrows refers to the malignant cells whereas blue arrows refers to necrotic areas (areas lacking eosinophilic structures).

Collagen is one of the most plentiful proteins in many living organisms which has been utilized as an effective and safe biomaterial in tissue engineering and clinical applications. Due to its biodegradability, biocompatibility, and weak antigenicity, it plays a significant role in biological structures^103^. Collagen-based nanoparticles are thermally stable, can decrease the systemic toxicity of drugs, and improve the uptake of nanoparticles by cells^104^. For the cancer in vivo studies, individual chemical medications, in some cases, do not effectively treat cancer. Therefore, combining collagen-NPs with such a chemical drug in a synergistic manner is a promising way to increase effectiveness. The impact of collagen-NPs and collagen-NPs/doxorubicin (DOX) in synergistic combination treatment on growth and apoptosis of Ehrlich ascites carcinoma (EAC) solid tumor has been examined in order to assess the in vivo role of collagen-NPs as an effective inducer of apoptosis (Fig. 13; Table 7). After 20 days of therapy, the average tumor volume in EAC mice (the control group) grew from 72.86 to 857.84 mm^3^. When collagen, collagen-NPs, and DOX were administered into EAC-bearing mice, tumor growth was considerably reduced by 50.85%, 71.21%, and 80.88%, respectively, in comparison to EAC control mice. Additionally, compared to mice treated with DOX and collagen-NPs separately, the collagen-NPs/DOX combination treatment showed considerable tumor growth suppression (95.58%). Collagen-NPs, DOX, and the combination of collagen-NPs/DOX were found to dramatically reduce the weight of tumor lumps in mice compared to EAC control mice. In contrast, the weight of tumor lumps in mice receiving the combination therapy was significantly lower (1.33 ± 0.17) as compared to mice receiving DOX (2.3 ± 0.31) or mice receiving collagen-NPs (2.91 ± 0.44) injections. The solid tumor volumes were roughly 50–100 mm^3^ (day 0) after five days of inoculation, prior to the start of the treatment, which meant even collagen nanoparticles or the commercial antitumor drug (DOX) did not reduce the tumour weight and volume, but they inhibited the acceleration in tumor growth. Since cancer cells show higher levels of free radicals compared with their normal counterparts^105^. As a natural polymer, collagen can release collagen peptides with higher bioactivity after enzymatic hydrolysis, which plays crucial roles in inhibiting lipid oxidation, scavenging free radicals, and helping maintain the balance of free radicals in human bodies, thereby reducing the risk of chronic non-communicable diseases^106^. So the nanoparticles of collagen have an inhibitory effect on the tumor cells.

**Table 7:** Inhibition effect of collagen-NPs, collagen, DOX, collagen + DOX & collage-NPs + DOX on EAC bearing mice tumor parameters.

Groups	Tumor weight (g)	Tumor volume average (mm^3^)		ΔT/ΔC (%)	Inhibition (%)
		0 day	20 days		
EAC	4.44 ± 0.4	72.86	857.84	100.0	0.0
Collagen	3.12 ± 0.55	77.66	423.9	44.1	55.89
DOX	2.3 ± 0.31	75.2	225.28	19.12	80.88
Collagen-NPs	2.91 ± 0.44	83	309.32	28.79	71.21
Collagen + DOX	1.9 ± 0.05	80.6	210.44	24.53	75.47
Collagen-NPs + DOX	1.33 ± 0.17	59.38	94.06	4.42	95.58

Hematoxylin and eosin-stained tumor section histopathological analysis (Fig. 13) in mice with an untreated tumor (control A), a large number of gigantic malignant cells (black arrows) with anaplasia, pleomorphism, nuclear dyschromasia, numerous atypical nuclei, and condensed chromosomes were seen to grow rapidly. Collagen-NPs (B) or DOX (C) treatment of mice bearing EAC resulted in decreased tumor growth rate, necrotic areas (areas lacking eosinophilic structures), noticeably more apoptotic bodies, and mild inhibition (blue arrows). In treating mice bearing EAC with both collagen-NPs/DOX (D), a strong favorable synergistic impact on the histopathological pattern was observed.

### Proposed mode of action

**Figure 14:**
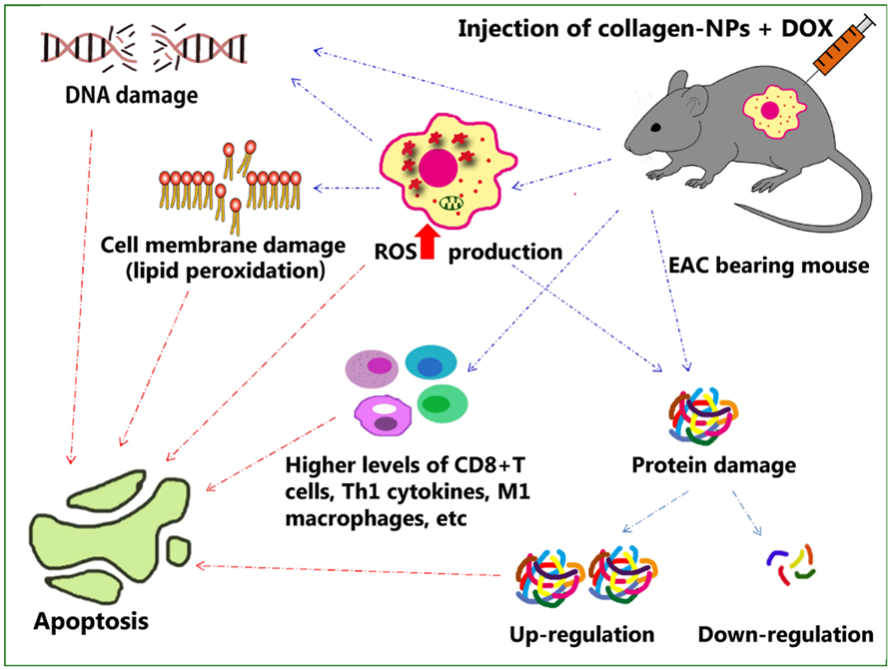
Schismatic diagram showing the proposed mode of action of the synergistic action between collagen-NPs produced by *Streptomyces xinghaiensis* NEAA-1 & DOX against EAC-bearing mice.

In general, nanoparticles cause tumor cells to apoptosis by several mechanisms, including transcription suppression, immunological interference, and reactive oxygen species (ROS)-mediated apoptosis (the most researched)^107^. Particularly because of their smaller and greater reactivity due to their higher surface area, nanoparticles are more effective at inducing apoptosis (Fig. 14). So, the synergistic combination of collagen-NPs and DOX can produce high levels of (ROS) which may alter the redox equilibrium of the cell. The most common apoptotic effects appear after elevation of tumor cells oxidative stress, followed by the release of inflammatory intermediates leading to DNA and protein damage^108^. More proteins are organized to reconfigure signaling and metabolic pathways in response to oxidative stress, which results in changes to the membrane and redox proteome shifts, which disrupt the cycle of progressing and proliferating and lead to apoptosis and suppressing tumors^109^. Bagheri et al.^110^ informed that the colloidal and physicochemical properties of nanoparticles allow for the suppression or generation of antibodies, supporting their widespread application in the detection and treatment of melanoma. In order to successfully regulate tumor cell proliferation, the other notion is to modify the immunosuppressive environment of the tumor with biocompatible nanoparticles that surround the dual compounds (like nucleic acid). According to the study of Mundekkad and Cho^107^, the dual therapeutic 5 triphosphate ds RNA (ppp dsRNA) encapsulated in nanoparticles stimulated higher levels of CD8^+^ T cells, Th1 cytokines, M1 macrophages, and other cells that are known to produce pro-inflammatory responses and significantly slow tumor growth.

### Antioxidant efficiency using ABTS^+^ radical scavenging

By inhibiting the oxidation of ABTS, collagen-NPs' antioxidant capabilities were assessed. ABTS^+^ radical solution was produced by reacting strong oxidizing agents (MnO_2_). The removal of cation colors (decolonization) by antioxidant compounds is the principle of the ABTS method, which donates hydrogen atoms to ABTS^+^ radicals. The results exhibited that collagen-NPs have a good antioxidant efficiency compared with collagen. The findings showed that 500 µg/mL collagen-NPs demonstrated 58% radical scavenging efficiency in a percentage inhibition manner with the comparison of the collagen (40.78%) and standard antioxidant ascorbic acid's activity of 89.6%. Collagen is a natural polymer that can release collagen peptides after enzymatic hydrolysis, which are characterized by higher bioactivity and play significant roles in lipid oxidation inhibition, free radicals scavenging, and assist in free radical’s balance maintenance in human bodies, reducing the risk of non-communicable chronic diseases^106^.

### Drug loading and encapsulation efficiency of methotrexate loaded collagen-NPs

**Figure 15:**
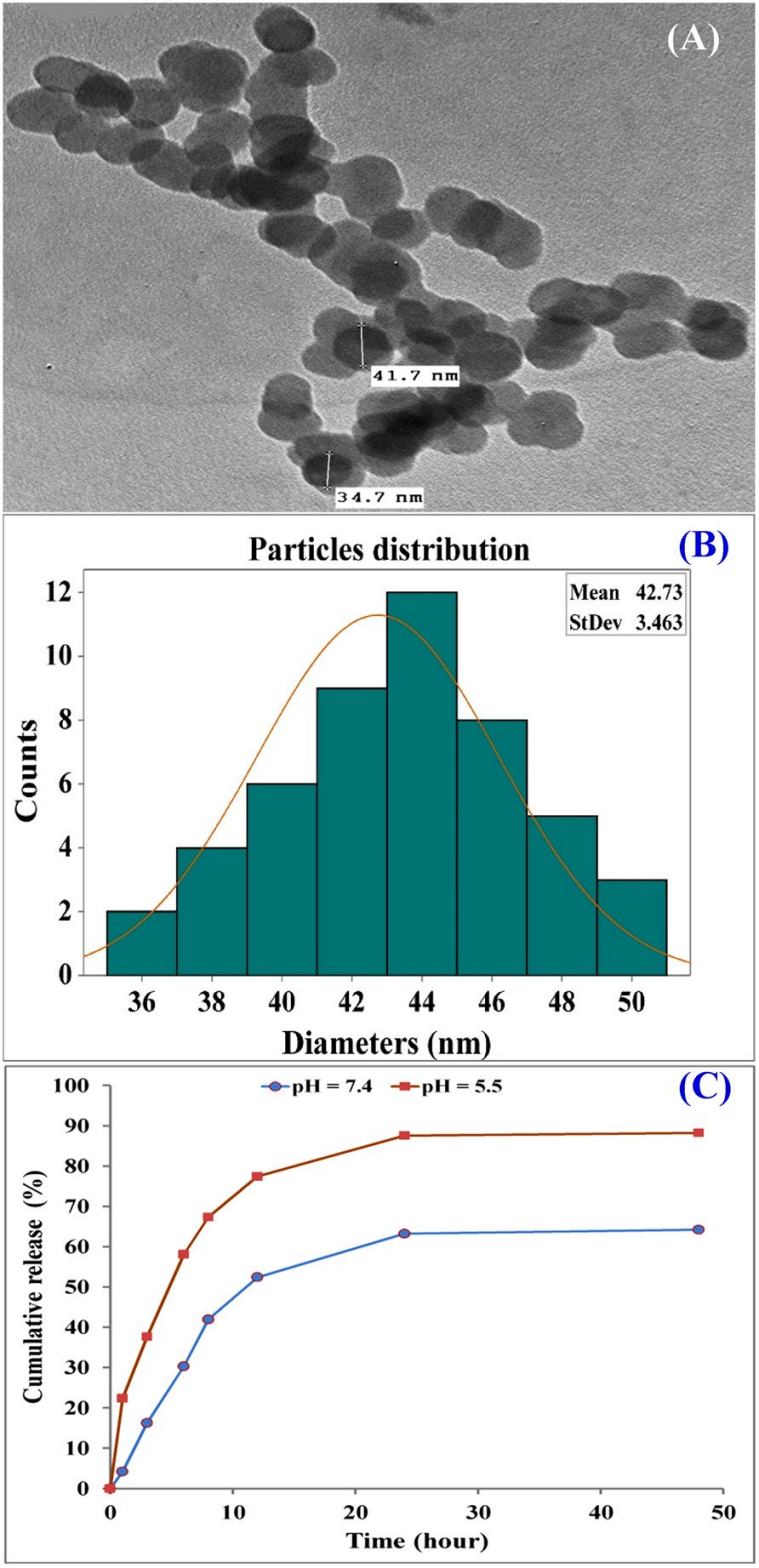
TEM image (**A**) of MTX loaded collagen-NPs, particles size distribution (**B**) and In vitro release of MTX loaded collagen-NPs (**C**).

The development of spherical nanoparticles was verified by TEM analysis of the shape of MTX-loaded collagen-NPs, as shown in Fig. 15A. The diameter distribution of MTX loaded collagen-NPs was also analyzed. Figure 15B shows the histogram obtained for MTX loaded collagen-NPs, where the average size calculated for MTX loaded NPs was 42.73 ± 3.5 nm, which exceeded the previous calculated average size of collagen-NPs (32.63 ± 14.59). The encapsulation efficiency (EE) was 48.91% and the drug loading (DL) was 24.45%, which was a satisfactory result given that the majority of existing nanomedicines had the disadvantage of having low drug-loading (often less than 10%), according to Shen et al.^111^ study.

### In vitro release of MTX loaded collagen-NPs

Figure 15C shows the cumulative amount of MTX released from the MTX-loaded collagen-NPs when the experiments were conducted at pH 7.4 and 5.5 PBS at 37 °C, respectively. After 1 h, there were two phases to the release of MTX from NPs, with a brief initial release of 8.23% and 22.24% at pH 7.4 and 5.5, respectively. After 12 h the release reached 30.34% and 77.44% at pH 7.4 and 5.5, respectively which consider slightly high rate of release at pH 7.4. This may be due to dissolution of collagen-NPs at pH 7.4 (isoelectric point of collagen). At the first 24 h, it appears that adsorbed MTX on the surface of the collagen-NPs showed high rate of separation from the NPs into the aqueous medium (the CR% were 40.24% and 87.56% at pH 7.4 and 5.5, respectively). Otherwise, at the next 48 h, the release rate was slower (the CR% were 41.22% and 88.22% at pH 7.4 and 5.5, respectively) due to continuous degradation and hydrolysis of collagen-NPs as a result of penetration of the aqueous media to the collagen-NPs previously informed by Maleki et al*.*^112^. The MTX release rate of the MTX loaded collagen-NPs at pH 5.5 revealed that the MTX release rate increased by decreasing pH, and a high value of the MTX release occurred in the first 24 h. The reason for that result may be due to acidic medium could affect the polymer (collagen-NPs) even by hydrolysis and degradation of the polymer matrix^113^ or could be due to the instability of NPs that tend to aggregate at acidic pH^114,115^. Due to tumor surfaces are highly acidic; this phenomenon reveals that the drug was selective to tumor tissue. Subsequently, MTX can easily be released from NPs in tumor cells medium and extracellular space tumor cells at a lower pH level.

## Conclusion

To the best of your knowledge, the current study is the first report on biological synthesis of collagen nanoparticles. The current study introduced an innovative eco-friendly approach for biosynthesis of collagen nanoparticles using *Streptomyces xinghaiensis* NEAA-1, which was consider is the most effective strain for collagen-NPs biosynthesis from newly isolated eight strains. The results are very promising. The characterization analyses confirmed the transformation of collagen to collagen-NPs and the efficacy of the cell free supernatant of *Streptomyces xinghaiensis* NEAA-1 as bio-reductant agent. The *in-vivo* and in vitro investigations proved the efficacy of collagen-NPs to be applied in various biomedical fields. In the present study, although the optimization of the bioprocess parameters was studied and maximized using FCCD, the large-scale production of collagen nanoparticles would be of great interest.

## Data Availability

All data generated or analyzed dusring this study are included in this article except the datasets that are available in the GenBank of The National Center for Biotechnology Information, [https://www.ncbi.nlm.nih.gov/nucleotide/OQ652077.1?report=genbank&log$=nucltop&blast_rank=1&RID=G68R6PDV013].
